# Multistep retrosynthesis combining a disconnection aware triple transformer loop with a route penalty score guided tree search†

**DOI:** 10.1039/d3sc01604h

**Published:** 2023-09-01

**Authors:** David Kreutter, Jean-Louis Reymond

**Affiliations:** a Department of Chemistry, Biochemistry and Pharmaceutical Sciences, University of Bern Freiestrasse 3 3012 Bern Switzerland david.kreutter@unibe.ch jean-louis.reymond@unibe.ch

## Abstract

Computer-aided synthesis planning (CASP) aims to automatically learn organic reactivity from literature and perform retrosynthesis of unseen molecules. CASP systems must learn reactions sufficiently precisely to propose realistic disconnections, while avoiding overfitting to leave room for diverse options, and explore possible routes such as to allow short synthetic sequences to emerge. Herein we report an open-source CASP tool proposing original solutions to both challenges. First, we use a triple transformer loop (TTL) predicting starting materials (T1), reagents (T2), and products (T3) to explore various disconnection sites defined by combining systematic, template-based, and transformer-based tagging procedures. Second, we integrate TTL into a multistep tree search algorithm (TTLA) prioritizing sequences using a route penalty score (RPScore) considering the number of steps, their confidence score, and the simplicity of all intermediates along the route. Our approach favours short synthetic routes to commercial starting materials, as exemplified by retrosynthetic analyses of recently approved drugs.

## Abstract 2

An efficient transformer-based retrosynthesis model, the triple-transformer loop algorithm (TTLA), is reported and proposes short routes from commercial building blocks for a variety of drugs.

## Graphical abstract

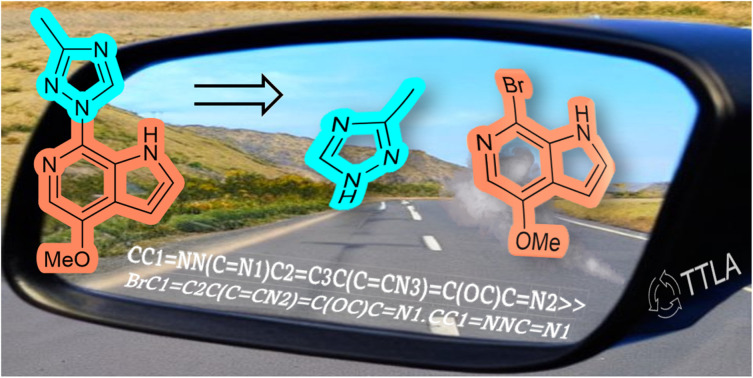

## Introduction

Retrosynthetic analysis consists in drafting a synthetic sequence to produce a desired product from available starting materials. This analysis is one of the most useful but also difficult tasks in organic chemistry because it requires to integrate the large and complex set of rules that have emerged from millions of reactions reported in almost 200 years of organic synthesis. Computer-aided synthesis planning (CASP), initially conceived by E. J. Corey in the 1960s,^1^ aims to harness the power of computers to automate retrosynthesis by exploiting data from experimental reactions collected in databases such as Reaxys^2^ or the open-access reaction dataset extracted from US patent office data.^3,4^ These databases list reactions of sets of starting materials (SM) and sets of reagents (R) to form one or several products (P).

While expert systems based on hand-written rules such as Chematica/Synthia™ perform quite well for synthesis planning,^5^ CASP ultimately aims to exploit artificial intelligence to automatically learn organic synthesis from reaction examples and propose synthetic routes for new molecules without human intervention.^6–10^ Template-based approaches extract reaction rules in the form of substructure transformations and use machine learning to learn their applicability domain from the structure of P in the training data.^11–14^ On the other hand, transformer-based models use the linear SMILES^15,16^ notation of chemical reactions and learn to translate the character string of P into the character string of SM + R, or *vice versa*.^17–27^ The single-step predictions are then iterated to propose multistep retrosyntheses of target molecules from a selected set of building blocks (BB), which requires prioritizing possible routes using search algorithms such as Monte Carlo tree search,^11,23,28^ and-or trees,^25,29^ or a multistep graph exploration.^24^

**Fig. 1:**
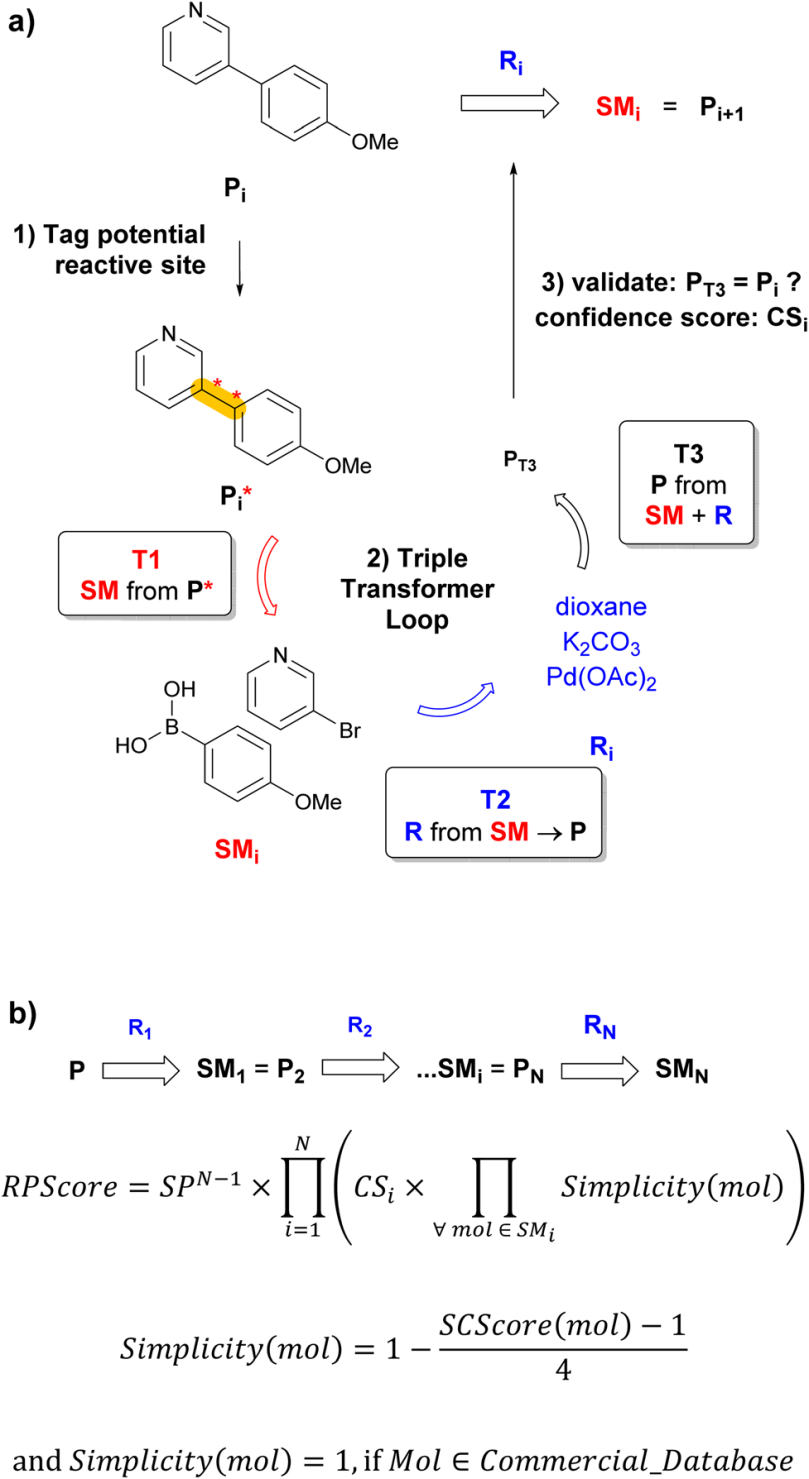
Multistep retrosynthesis using TTLA. (a) Single-step retrosynthesis. At step i, each potential reactive site in P_i_ is identified systematically, using templates or a tagging transformer, and labelled to produce 
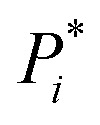
. Transformer T1 is applied to 
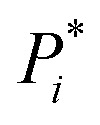
 to predict SM_i_ (one or more starting materials), transformer T2 is applied to the top-scoring SM_i_ → P_i_ to predict R_i_ (one or more reagents), and finally transformer T3 is applied to the top-scoring SM_i_ + R_i_ to produce P_T3_. The prediction is finally validated if P_T3_ = P_i_ with confidence score CS_i_ of T3. Each molecule in the SM_i_ set is then used as product P_i+1_ for the next iteration. The route branches out if SM_i_ contains multiple molecules. (b) TTLA sequence and route penalty scoring. All molecules in the SM_i_ set of each step are used in the RPScore calculation of a linear sequence. See text for details.

Any CASP system must overcome two critical challenges to propose realistic retrosyntheses. First, the system must learn the context of reactions sufficiently well to propose reactions that make sense, but without overfitting such as to propose diverse retrosynthetic operations on previously unseen molecules. Second, the route-prioritizing algorithm must be designed to allow short sequences to emerge from the multitude of predicted possibilities.^6^ Herein, we report a transformer-based retrosynthesis tool which proposes original solutions to both challenges. For single-step retrosynthesis, we use three different transformer models assembled as a triple transformer loop (TTL, Fig. 1a). To broaden the scope of predicted disconnections on a given target molecule, the TTL explores multiple disconnections by using products with tagged reaction centers (P*) obtained by combining systematic, template-based and transformer-based tagging procedures. Compared to a transformer model trained on predicting SM + R directly from P*, the TTL achieves better round-trip accuracy for single-step retrosynthesis. For multistep retrosynthesis predictions, we integrate the TTL into a multistep tree search algorithm, here named TTLA, which selects reaction sequences using a new route penalty score (RPScore), which for a route of N steps, is the product of a step-penalty score SP^N^, the confidence scores of each single-step retrosynthesis (CS), and the simplicity scores^24^ of all SM along the route (Fig. 1b). This selection scheme favours short sequences and is exemplified with the prediction of synthetic routes for recently approved drugs.

## Methods

### Dataset

The United States Patent and Trademark Office (USPTO) chemical reaction dataset version shared by Thakkar *et al.*^30^ was used for the single-step evaluation as well as for training all transformer models in this study. The dataset is derived from the version of Lowe^3,4^ and has been filtered by these authors to include reactions with a single product (P) and between 2 and 10 starting materials (SM) and reagents (R) only. In the present work, we removed the tagging information, and reactions were remapped and retagged using our new SMILES tagging strategy and syntax. The same dataset split for training, validation, and test (90 : 5 : 5), as shared by Thakkar *et al.*^30^ was used across all models resulting in 1 139 608, 63 672 and 63 454 reactions respectively.

### Tagging reaction centers

Training the disconnection-aware retrosynthesis model requires a training dataset where all product SMILES have tagged atoms. To tag reacting atoms, we use the atom-mapping tool shared by Schwaller *et al.*^31^ to identify the atoms having an environmental change during the reaction, defined as reacting atoms. These reacting atoms are then re-labelled with the atom mapping label “1” while all other atom mapping labels are removed, as described by Byekwaso *et al.*.^32^ We then replace the atom tagging syntax by its unmapped SMILES notation, *e.g.* replacing “[C:1]” with “C”, and append the atom with another separated tagging token (“!”) using RDkit.^33^ This modification allows to maintain an invariant SMILES token usage independent of the neighbouring hydrogen count or stereochemistry.

### Single-step disconnection aware retrosynthesis (T1)

Being able to identify the reaction center of a given reaction, we apply our reaction tagging algorithm on USPTO to obtain a retrosynthesis-tagged training dataset. We remove reagents, catalysts, and solvents, which are identified as the unmapped species in atom-mapped reactions and train the retrosynthesis model to predict the starting materials given as input the tagged product. We use the transformer architecture^18^ and train it using the OpenNMT^34,35^ library with standard previously-reported hyperparameters for this type of task.^22^

### Automatic tagging of potentially reactive atoms

We use three complementary methods to maximize the tagging possibilities while maintaining a reasonable number of predictions. First, we systematically tag all possible single atoms, pairs of directly connected atoms, and triplets of adjacent atoms (chain or three-membered ring). Secondly, we use templates for tagging the reactive sets of atoms corresponding to the conditional substructure with a variable radius (typically from 1 to 3). Templates occurring more than once and having between 1 and 10 reactive atoms were identified by analyzing the original USPTO dataset. A given template can contain multiple disconnected sets of reactive atoms. Finally, the transformer model AutoTag reported by Thakkar *et al.*^30^ was trained from untagged SMILES to the corresponding tagged molecule to provide additional tagging examples.

### Reagent prediction (T2)

Transformer T2 is trained from the untagged USPTO training set to identify reagents (R) from the combination of SM and P using the same hyperparameters as for T1. Note that R often includes actual reagents and solvents.

### Forward validation (T3)

The third model of the triple-transformer loop is a forward reaction prediction model trained with untagged reactions (molecular transformer).^22^ We give this forward validation model the predicted SM_i_ (from T1) and the predicted R_i_ (from T2) as input separated by the “>” token. If T3 predicts the correct P_i_ as its top-1 prediction, those SM_i_ and R_i_ are stored for the tree search. The confidence score CS_i_ for the T3 prediction is used as confidence score for the reaction. T3 serves to filter down a large number of predictions to retain feasible reactions only.

### Single-step TTL tagging strategies study

The performance of individual tagging methods was studied on 500 molecules randomly selected from the USPTO test set for single-step TTL retrosynthesis to which we varied the three strategies over various parameters, changing the template radius from 1 to 3 and the transformer tagging (AutoTag) beam size from 1 to 1000.

### Route penalty score (RPScore)

The RPScore is computed for each predicted linear retrosynthetic sequence of N steps leading from the final product P to starting materials SM_N_ (Fig. 1b). To reduce the score of long sequences, we introduce a step penalty SP, with 0 < SP ≤ 1, extended to SP^N^ for a sequence of N steps. The RPScore is the product of SP^N^ with the product of all confidence scores CS_i_ (from the T3 prediction) for each individual step and the Simplicity (mol) for all intermediates along the sequence of N steps. By default, the penalty value SP is set to 0.8, but this could be adapted for every search in the configuration file of the multistep exploration. Simplicity(mol)^24^ ranges from 0 for complex to 1 for simple molecules and is derived from the molecular synthetic complexity score (SCScore, ranging from 1 to 5) which describes molecular complexity taking synthetic accessibility into account.^36^ Here, we assign a value of 1 if the molecule occurs in the BB set of commercial starting materials. In contrast to Schwaller *et al.*,^24^ we exclude reagents R_i_ from the Simplicity calculation to avoid penalizing steps that use reagents with low calculated Simplicity, which is rarely a measure of their availability or ease of use.

### Multistep exploration strategy

We use a Heuristic Best-First Tree Search algorithm with beam search and iterative expansion to explore retrosynthetic routes as similarly reported for transformer-based retrosynthesis.^24^ Once predictions of an iteration are complete, the tree search updates and lists all possible routes, and computes the RPScore. Unsolved routes are sorted by decreasing RPScore. The top 20 unsolved routes, which lead to starting materials absent from the selected set of commercially available building blocks, are selected for expansion by defining them as products P_i_ and new SM_i_ are predicted by applying a single-step retrosynthesis using TTL. The resulting set of predicted single-step retrosynthesis is updated back to the tree wherever those SMs were present. The tree is updated for the next iteration. The process stops when a chosen minimum number of solved routes or a maximum number of iterations has been reached.

### Building block (BB) set

We combined MolPort (https://www.molport.com) and Enamine (https://www.enamine.net) databases to build our database of 534 058 commercially available compounds as the building block (BB) set.

## Results and discussion

### Training transformer T1 for single-step retrosynthesis

Initially, we use the atom-mapping transformer^31^ information to annotate reacting atoms in all products P in the training data, which results in a training dataset containing labelled P*. Our code is inspired by the recent report by Byekwaso *et al.*,^32^ however with a slightly simplified syntax for tagged atoms. Using the tagged P* data, we then train a transformer model T1 to predict SM from P*, a task which is simpler than predicting both SM and R from P* as done by Byekwaso *et al.*^32^

### Tagging potential reactive sites

**Fig. 2:**
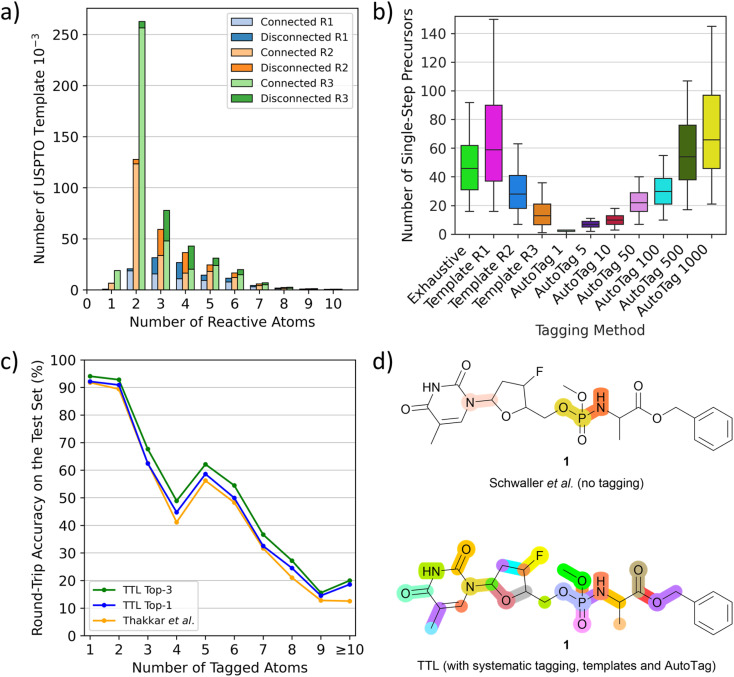
TTL and automatic atom tagging. (a) Distribution of the number of tagging templates extracted from USPTO depending on the number of atoms it tags, named “reactive atoms”. Triple bar plot to show the differences between conditional radiuses beyond tagged atoms from 1 to 3 (R1 to R3). Bars are split into a light-coloured part representing the fraction of templates that tags bond-connected atoms and dark-coloured for disconnected atoms. (b) Number of validated single-step starting materials (“precursors”) on the TTL generated depending on the automatic tagging strategy, tested over 500 molecules randomly selected from the TTL test set. (c) Round-trip accuracies of the TTL using the top-1 SM by T1 and the top-1 or top-3 R predicted by T2, compared to the disconnection-aware dual transformer of Thakkar *et al.*^30^ (d) Highlighted disconnection sites of the antiviral molecule 1 using the untagged retrosynthesis and forward validation models of Schwaller *et al.*,^24^ leading to four unique sets of starting materials among three reactive sites (top) and the TTL augmented by systematic tagging, template-based tagging (radius 2) and AutoTag (beam size 50) after forward validation leading to 231 unique sets of starting materials among 26 reactive sites (bottom).

To use T1 to predict possible SM_i_ from a given product P_i_ at step i, one must first tag potentially reacting atoms in P_i_. We do this using complementary methods. First, we tag all single atoms as well as pairs and triplets of adjacent atoms systematically in P_i_. Second, we systematically apply templates extracted from tagged P* in the USPTO dataset. These templates with various conditional radiuses (from 1 to 3) are substructures containing up to ten tagged atoms, not necessarily connected. Although the most frequent templates are those with two or three connected atoms, which are tags also obtained in the systematic procedure, the templates also include many tags with disconnected atom pairs and triplets as well as tags with four or more atoms, which are missing from the systematic tagging procedure (Fig. 2a). As a third tagging option, we use the tagging approach recently reported by Thakkar *et al.*^30^ where reacting atoms are identified using a tagging transformer, here named AutoTag, trained to learn the detailed context from the tagged dataset. The number of predicted tags (sorted by confidence score, called beam size) of AutoTag can be varied to generate a given number of possible tags to extend the retrosynthesis options.

Analyzing the performance of the different tagging methods shows that less restrictive template radius or high AutoTag beam size both lead to an increased number of tagged atoms per molecule (Fig. S1†) as well as a much higher number of generated tagged SMILES (Fig. S2†) and significantly more single-step starting materials (Fig. 2b and S3†), but also to a lower number of high confidence predictions (Fig. S4†), indicating that most of the additionally obtained tags are less chemically meaningful (Fig. S5†). Moreover, the tagging efficiency, evaluated by dividing the number of successful retrosynthetic steps obtained by the number of TTL rounds (number of tags), drops for high AutoTag beam sizes and low radius templates (Fig. S6†). To obtain a good number of validated retrosynthetic steps at reasonable computing cost, we combine three strategies: the systematic tagging (1, 2 and 3 atoms), templates with a radius of 2, and the AutoTag transformer with a beam size of 50. A Venn diagram analysis of the number of unique retrosynthetic steps obtained shows that 17% of the steps (37.8% of high confidence steps) are predicted by all three methods, while 52.6% of the steps (25.5% of high confidence steps) are coming from only one of the three tagging methods, highlighting their complementarity (Fig. S7 and S8†).

### Triple transformer loop (TTL)

To initiate a validated single-step retrosynthesis prediction for product P_i_, we run T1 on all P^*^_i_ obtained by the combined selected tagging procedures described above. The transformer outputs a series of possible SM_i_, which are sorted in order of the T1 confidence score. For the top-*B* SM_i_ (beam size *B* = 1 or more), we then apply a second transformer (T2) trained to predict R from SM → P. For each SM_i_, T2 outputs a series of possible R_i_, from which we retain the top-*B*′ (beam size *B*′ = 1 or more). The TTL is completed with a forward validation^37^ transformer (T3) trained to predict P from SM + R using the same training dataset used for T1 and T2. For all combinations of top SM_i_ predicted by T1 and top R_i_ predicted by T2, we finally use T3 to predict the most likely product P_T3_. The TTL prediction is validated if the top-1 predicted P_T3_ is identical to the input product P_i_ (Fig. 1a). The T3 confidence scores CS_i_ of the validated predictions SM_i_ + R_i_ are used to select the best R_i_ if *B*′ > 1, and to calculate the route penalty score (RPScore, see below).

### Performance evaluation

The performance of TTL can be compared with previous single-step retrosynthesis models at three different levels. First, transformer T1, which predicts SM from the tagged product P*, can be compared with other single-step retrosynthesis models predicting SM from P, both transformer-based and template-based.^17,19,21,23–27^ While these models perform between 40% and 55% top-1 accuracy, our tagged T1 achieves 66% top-1 accuracy, which shows that tagging provides a significant advantage for this task.

Second, the performance of the TTL loop can be compared with the disconnection-aware retrosynthesis model of Thakkar *et al.*^30^ in terms of single-step round-trip prediction accuracy from the tagged product P*, which is the accuracy of predicting P from the SM + R initially predicted from P*. TTL using only the top-1 predictions for T1 and T2 performs comparably to Thakkar's disconnection-aware retrosynthesis model (80.44% *vs.* 79.09% accuracy). The TTL performance increases to 83.04% when considering the top-1 prediction of T1 and the top-3 predictions of T2. Similar to the observation by Thakkar *et al.*,^30^ we furthermore find that the prediction accuracy strongly decreases as a function of the number of tagged atoms (Fig. 2c). Subsequently to our preprinted report, a separate study has investigated the performance of the reagent prediction transformer.^38^

Thirdly, one can compare the single-step round trip accuracy of TTL with that of the non-tagged retrosynthesis model of Schwaller *et al.*,^24,37^ who evaluated if a forward prediction model predicted the correct product P from the SM + R predicted by their model from the non-tagged P. As discussed by Thakkar *et al.*,^30^ the untagged transformer may sometimes choose a different and easier to predict disconnection than that recorded in the test set, and therefore performs slightly better (82.4% top-1 accuracy) than the tagged transformer, which is forced by tagging to apply the retrosynthesis of the test set. Here, we find that the top-1 round-trip prediction accuracy (P → P), obtained by applying our multiple tagging procedure followed by the TTL, reaches 99.9%, which means that our approach is almost always able to propose at least one forward-validated possible retrosynthetic step from any product molecule.

Furthermore, a critical feature of any single-step retrosynthesis model in view of multi-step retrosynthesis concerns the diversity of possible disconnections proposed. We find that this diversity is greatly enhanced by the multiple tagging approach. For instance, when tested on unseen molecules, the TTL combined multiple tagging provides validated disconnections at several possible reactive sites. By contrast, the baseline transformer, trained as reported by Schwaller *et al.*^24^ to produce SM directly from P using the unannotated data for training, chooses fewer disconnection points, as exemplified here for the pro-nucleotide 1 (Fig. 2d).^39^

### Multistep retrosynthesis

By integrating the single-step retrosynthesis TTL into a multistep tree search, we obtain a multistep retrosynthesis algorithm, here named TTLA. In each retrosynthesis iteration, TTLA runs the TTL exhaustively on all SM of the preceding iteration, newly defined as P, and ranks the routes to the newly predicted SM using a composite route penalty score RPScore (Fig. 1b, see Methods for details).

When prioritizing multiple retrosynthesis options during the tree search, TTLA uses the RPScore to rank the different routes leading to the SM produced in the latest iteration of TTL, and only extends retrosynthesis on a small number (typically 20) of SM taken from the top RPScoring routes. Because each additional step imposes a penalty (usually P = 0.8), lengthy routes and unproductive loops involving protection/deprotection cycles of the same functional group are rapidly falling down the RPScore priority list, which leads the algorithm to explore alternative routes, so that short synthetic sequences are eventually prioritized even if their first retrosynthetic steps were initially not top scoring.

As commonly observed with CASP tools as well as with transformer models in general, the top-scoring outputs of TTLA must be inspected to identify relevant predictions. While the RPScore is used in the tree search, we find relevant routes by inspecting both the top-RPScoring route and the top-CScoring routes (CScore (route) = the product of CS_i_ for all steps) in the TTLA output, as discussed below with examples.

**Fig. 3:**
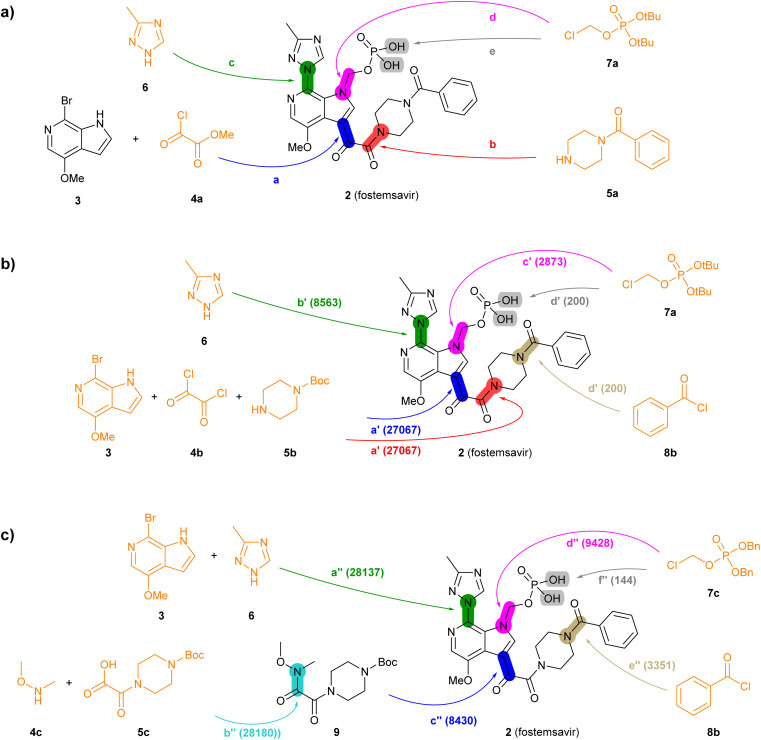
Summary of reported and TTLA predicted routes for fostemsavir 2. Bonds formed in each step are highlighted in colour. Numbers in parenthesis correspond to the order in which the multistep tree search prioritized predictions. The full retrosynthesis routes are drawn out in the ESI† Fig. S9–S11.† (a) Commercial process. Reported reagents: (a) AlCl_3_, Bu_4_NHSO_4_, CH_2_Cl_2_, then KOH, then H_3_PO_4_; (b) Ph_2_POCl, NMM, NMP; (c) KOH, CuI, then KOH, EtOH, LiI; (d) Et_4_NI, K_2_CO_3_, CH_3_CN/H_2_O; (e) AcOH, H_2_O. (b) Highest TTLA RPScoring route. Predicted reagents: (a′) Et_3_N, CH_2_Cl_2_; (b′) K_2_CO_3_, CuI, toluene; (c′) K_2_CO_3_, DMF; (d′) HCl, *N*,*N*-diisopropylethylamine, H_2_O, dioxane. (c) Highest TTLA CScoring route. Predicted reagents: (a′′) (2S)-pyrrolidine-2-carboxylic acid, K_2_CO_3_, CuI, EtOAc, DMSO; (b′′) no reagent predicted; (c′′) *n*-BuLi, THF; (d′′) K_2_CO_3_, DMF; (e′′) TFA, DMAP, CH_2_Cl_2_, (f′′) Pd, EtOH.

**Fig. 4:**
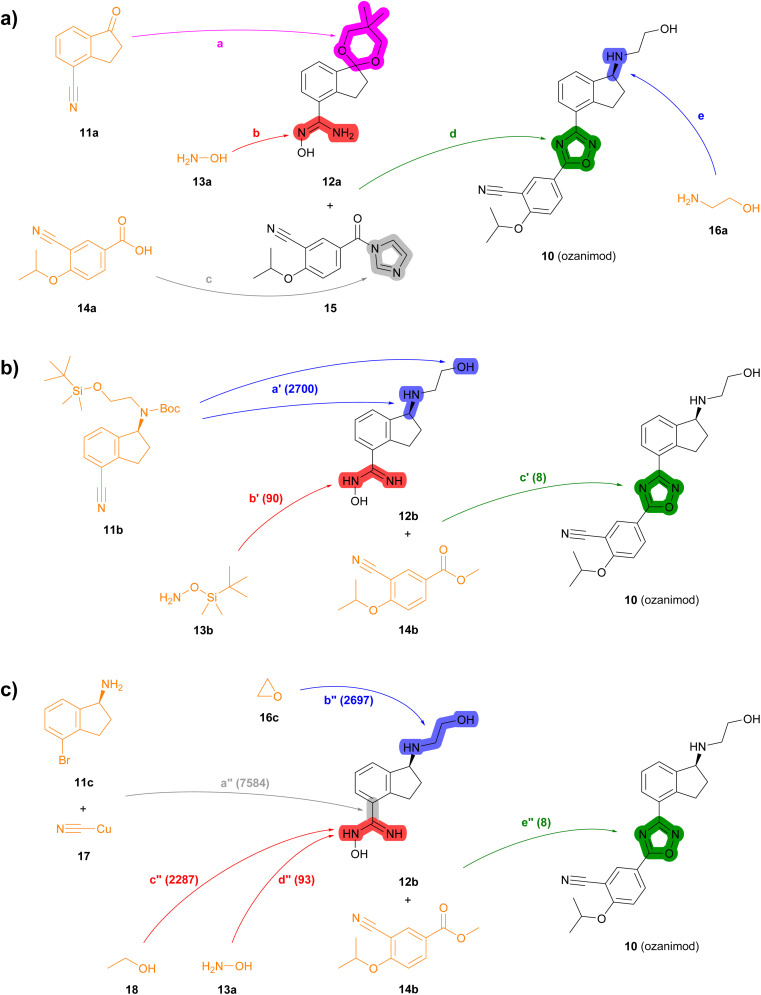
Summary of reported and TTLA predicted routes for ozanimod 10. Bonds formed in each step are highlighted in colour. Numbers in parenthesis correspond to the order in which the multistep tree search prioritized predictions. The full retrosynthesis routes are drawn out in the ESI† Fig. S12–S14.† (a) Commercial process. Reported reagents: (a) HC(Ome)_3_, *p*-TsOH, PhCH_3_; (b) NH_2_OH·HCl, Et_3_N; (c) carbonyl diimidazole; (d) NaOH; (e) (i) *p*-TsOH, acetone, (ii) NH_2_CH_2_CH_2_OH, *p*-TsOH, PhCH_3_, (iii) chiral Ru-complex, Et_3_N/HCO_2_H. (b) Highest TTLA RPScoring route. Predicted reagents: (a′) HCl, dioxane; (b′) ZnCl_2_, AcOEt, toluene; (c′) HCl, *t*-BuOK, THF. (c) Highest TTLA CScoring route. Predicted reagents: (a′′) 1-Methylpyrrolidin-2-one; (b′′) no reagent predicted; (c′′) HCl, Et_2_O; (d′′) HCl, NaHCO_3_, EtOH; (e′′) HCl, *t*-BuOK, THF.

TTLA is exemplified here for predicting the synthesis of two drug molecules approved in 2020, namely fostemsavir (2, Fig. 3), a prodrug which upon phosphatase cleavage releases the antiretroviral agent temsavir as HIV entry inhibitor,^40^ and ozanimod (10, Fig. 4), a sphingosine-1-phosphate receptor antagonist used as an immunomodulatory agent to treat multiple sclerosis.^41^ The commercial process for both drugs was recently reviewed.^42^ None of the synthetic steps involved in these two processes occur in the USPTO dataset used for training TTL, making them a good test case for TTLA. For these examples, we challenged TTLA to predict synthetic routes starting from a list of 534 058 commercially available BB.

The reported commercial process for the antiviral drug fostemsavir (2, Fig. 3a, details in Fig. S9†) is a linear sequence involving the sequential *C*-acylation of pyrrolopyridine 3 with oxalyl monochloride 4a (step a) and benzoylpiperazine (5a, step b), followed by coupling of with triazole 6 (step c), *N*-alkylation of the pyrrole with the protected chloromethylphosphate 7a (step d), and finally deprotection of the *tert*-butyl ester protecting groups (step e).

When challenged with 2, TTLA proposes many possible routes from similar starting materials as the commercial process, but in a different order. The highest RPScoing route is a linear sequence starting from the double *C*- and *N*-alkylation of oxalyl chloride (4b) with pyrrolopyridine (3) and 1-boc-piperazine (5b) in one pot (step a′, Fig. 3b, details in Fig. S10†). The aryl bromide of the resulting intermediate is then substituted with triazole 6 (step b′), and its pyrrole NH group is alkylated with *tert*-butyl chloromethyl phosphate 7a, similarly to the commercial route (step c′). In the final step, the phosphate and the piperazine groups are deprotected with acid, followed by benzoylation of the free piperazine with benzoylchloride (8b) to form fostemsavir 2 (step d′).

On the other hand, the highest CScoring route is a convergent sequence starting with alkylation of triazole 6 with pyrrolopyridine 3 on the one hand (step a′′, Fig. 3c, details in Fig. S11†), and the preparation of the Weinreb amide 9 from boc-oxalylpiperazine 5c and *N*,*O*-dimethylhydroxylamine 4c on the other hand (step b′′). The resulting intermediates are then coupled (step c′′), and the product is *N*-alkylated on the pyrrole nitrogen with benzyl-protected chloromethyl phosphate 7c (step d′′). Deprotection of the piperazine group allows the acylation with benzoylchloride (8b, step e′′). Reductive deprotection of the benzyl phosphate esters finally gives the product 2 (step f′′).

In the second example, the drug ozanimod 10 is synthesized commercially in a convergent sequence of 7 steps from ketone 11a and benzoic acid 14a (Fig. 4a, details in Fig. S12†). After initial protection of ketone 11a as an acetal (step a), its nitrile group is reacted with hydroxylamine 13a to form the *N*-hydroxyamidine intermediate 12a (step b). In parallel, benzoic acid 14a is activated to the corresponding benzoyl imidazole 15 (step c). Intermediates 12a and 15 are then condensed to form the oxazole ring (step d). The acetal group of the resulting intermediate is then deprotected and condensed with ethanolamine (16a) to the corresponding imine, which is reduced enantioselectively using a chiral ruthenium catalyst to form 10 (step e).

Many of the high-scoring routes identified with TTLA are extremely short sequences starting with commercially available close analogs of the drug and were removed from the list of top-scoring routes. Interestingly, TTLA also proposes routes that resemble the commercial process but start from chiral starting materials such as aminoindanes 11b and 11c, which avoids the enantioselective reaction used for the commercial process. For example, the best RPScoring route is a linear synthesis from 11b starting with the removal of the Boc and TBS protecting groups of the ethanolamine side chain and conversion of the cyano group to the corresponding *N*-hydroxyamidine by reaction with TBS-hydroxylamine (13b) to form intermediate 12b (steps a′ and b′, Fig. 4b, details in Fig. S13†). The third and final step of this short sequence is the condensation of *N*-hydroxyamidine 12b with cyanobenzoate 14b yielding ozanimod 10 (step c′).

The best CScoring route is a somewhat longer linear sequence employing the same condensation of 12b and 14b as the final step (step e′′, Fig. 4c, details in Fig. S14†). In this proposed sequence however, intermediate 12b requires four steps from the chiral aminobromoindane 11c as follows. First, the cyano group is installed by reaction of the aryl bromide with copper cyanide (step a′′). Second, the primary amine reacts with ethylene oxide 16c to form the *N*-hydroxyethyl side chain (step b′′). Third, the cyano group introduced in step a′′ reacts with ethanol (18) to form an ethyl imidate intermediate (step c′′), which further reacts with ethanolamine (13a) in a fourth step to form the *N*-hydroxyamidine group in 12b (step d′′).

Analyzing the details of the TTLA collective output shows that, although TTLA did not formulate routes identical to the commercial processes, the set of commercial starting materials used by TTLA are very similar to those used in the reported commercial processes for both drugs (Fig. S15 and S16†). In fact, all starting materials used in the commercial process for fostemsavir are present in the set for this drug.

In terms of individual reaction steps, we find that TTLA explores a large number of single reactions to arrive at the top-scoring short routes proposed in the above retrosynthesis. In the case of fostemsavir, the key retrosynthetic *C*- and *N*-acylation of the oxalyl starting material is discovered after 27 067 single predicted steps (Fig. 3b, step a′), probably because this step is rather complex and unusual. In the case of ozanimod, TTLA performed 7594 individual single-step predictions to arrive at the proposed retrosyntheses, with the best scoring route being discovered after 2700 iterations. Interestingly, the formation of the oxadiazole ring is discovered already at iteration 8 (Fig. 4b, step c′). It should be noted that the order of iterations and therefore the number of attempts necessary to identify high-scoring routes depends on the scoring function used to prioritize node expansion, here the RPScore, which takes the simplicity and number of steps into account.

**Fig. 5:**
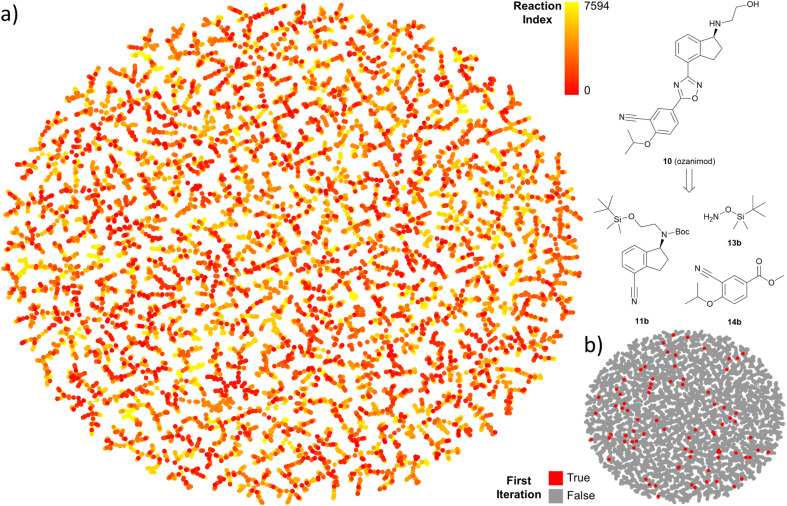
TMAP representation of iterated predictions for the multistep search of ozanimod. (a) Predicted reactions from the target molecule (low indexes) to end nodes. (b) Highlighted first iteration of the TTLA search. Interactive map available at https://tm.gdb.tools/TTLA/ozanimod.

The output of TTLA can be visualized by representing the collective predicted single steps in a TMAP^43^ computed using the differential reaction fingerprint (DRFP)^44^ as a similarity measure. As illustrated for ozanimod, colour-coding by step iteration number indicates that TTLA explores a broad diversity of steps directly from the beginning of the retrosynthesis exploration, which we attribute to our diverse reaction center tagging approach used (Fig. 5a). This diversity is also visible when colour-coding all steps involving the final product, corresponding to the initial retrosynthesis, which are broadly distributed on the map (Fig. 5b). A similar pattern is visible in the TMAP of the predicted single steps for fostemsavir (Fig. S17†).

### Comparing TTLA with other retrosynthesis tools

Previous retrosynthesis tools, template-based or transformer-based, predict starting material from products by applying the most probable retrosynthetic operation according to a training set. Here we combined exhaustive and template-based methods to label many potential reactive sites, which lead us to test many possible disconnections (Fig. 2d). These potential reactive sites were then challenged with the TTL, which produced detailed predictions including starting materials and reagents. In the examples discussed above TTLA identified short routes comparable to the reported processes, which were all examples of optimized production routes.

By comparison, a currently available version of AiZynthFinder (v3.7.0),^14^ a templated-based retrosynthesis tool, fails to propose a synthesis for fostemsavir due to its inability to find a synthesis for a bis-*tert*-butyl phosphate starting material (Fig. S18†). AizynthFinder furthermore proposes a short route similar to TTLA for ozanimod, although including somewhat less realistic steps, for example, an alkylation of a primary amine with 2-bromoethyl acetate which would probably rather lead to acetyl transfer, and no indication of reagents (Fig. S19†). On the other hand, the online portal of IBM RXN for chemistry,^45^ which uses a transformer model, predicts essentially the same route as TTLA for fostemsavir (Fig. S20†). For ozanimod however, this tool settles on an eight-step route which, although containing realistic steps, is simply much longer than the commercial process or the route proposed by TTLA (Fig. S21†). For both of these retrosynthesis tools, whether the routes are part of their training sets is not known.

To statistically evaluate our TTLA, we selected target molecules from the retrosynthesis benchmark dataset shared by Genheden *et al.* which were absent from our training dataset.^46^ Due to the high computing time of our method, a random subset of 240 target molecules was selected. Solved routes involving reaction steps present in our training dataset were removed from the evaluation. TTLA proposed retrosyntheses to commercially available starting materials for 97.5% of the target molecules, which is comparable to the performance of other retrosynthetic tools reported in the original paper.^46^ Selected examples are shown in Fig. S22–S31.†

## Conclusion

In summary, our data shows that a triple transformer loop (TTL) operating on products with tagged reactive atoms achieves efficient single-step retrosynthesis predictions. TTL was integrated into a tree-exploration strategy using a route penalty scoring scheme to form the multistep retrosynthesis tool TTLA, which can predict short synthetic routes for drug molecules. Since our approach uses transformer models, it should be possible to specialize TTLA for specific reaction classes by transfer learning similar to transformer models for forward prediction.^47^ Furthermore, predicting SM from P and R from SM + P separately might be potentially adapted to reactions with more complex reagents such as enzymes^48–50^ and help expand the scope of CASP systems. It should however be noted that the use of multiple transformer models and the detailed analysis of many possible disconnections renders our approach relatively slow, requiring up to several hours of computing time for a full retrosynthetic analysis. Efficiency increases might be possible in the future by fine-tuning the selection of potential disconnections and improving the tree search.

## Data availability

Code and instructions to compute multistep retrosynthesis as well as the code to tag reactive sites can be found on our GitHub repository: https://github.com/reymond-group/MultiStepRetrosynthesisTTL. The original USPTO dataset can be found at https://doi.org/10.6084/m9.figshare.5104873.v1. The derived version of USPTO of Thakkar *et al.* could be found in their Zenodo repository.^30,51^

## Author contributions

DK designed and carried out the study and wrote the paper, JLR designed and supervised the study and wrote the paper.

## Conflicts of interest

The authors declare that they have no competing interests.

## Supplementary Material

SC-014-D3SC01604H-s001
