# Supplementary material for: Multistep retrosynthesis combining a disconnection aware triple transformer loop with a route penalty score guided tree search
Source: Chem Sci. 2023 Sep 1;14(36):9959–69. doi: 10.1039/d3sc01604h (PMC10510629; doi:10.1039/d3sc01604h)
Supplement: SC-014-D3SC01604H-s001 [file SC-014-D3SC01604H-s001.pdf]

Electronic Supplementary Material (ESI) for Chemical Science.
This journal is © The Royal Society of Chemistry 2023

Supporting Information for

Multistep retrosynthesis combining a disconnection aware triple transformer loop with a route penalty score guided tree search

Single-step tagging strategies study

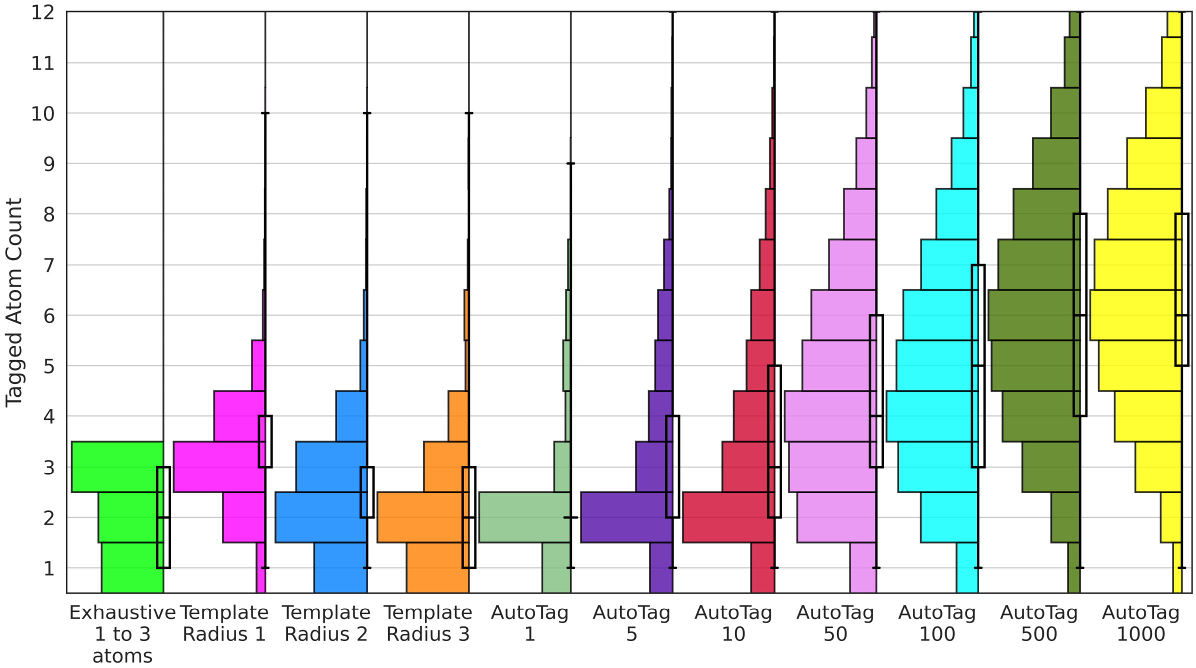

Figure S1. Number of tagged atoms per molecule as function of the tagging method. The relative number of molecules (horizontal bar length) is plotted as function of the number of atoms tagged (vertical axis) by different tagging methods (horizontal categories), tested over 500 molecules (randomly selected from the test set). The exhaustive tagging was performed together for tags containing 1, 2 and 3 atoms. The template tagging was performed separately for templates of radius of 1, 2 or 3 bonds. The AutoTag model was tested using the top-B” predictions using B” = 1, 5, 10, 50, 100, 500 and 1000.

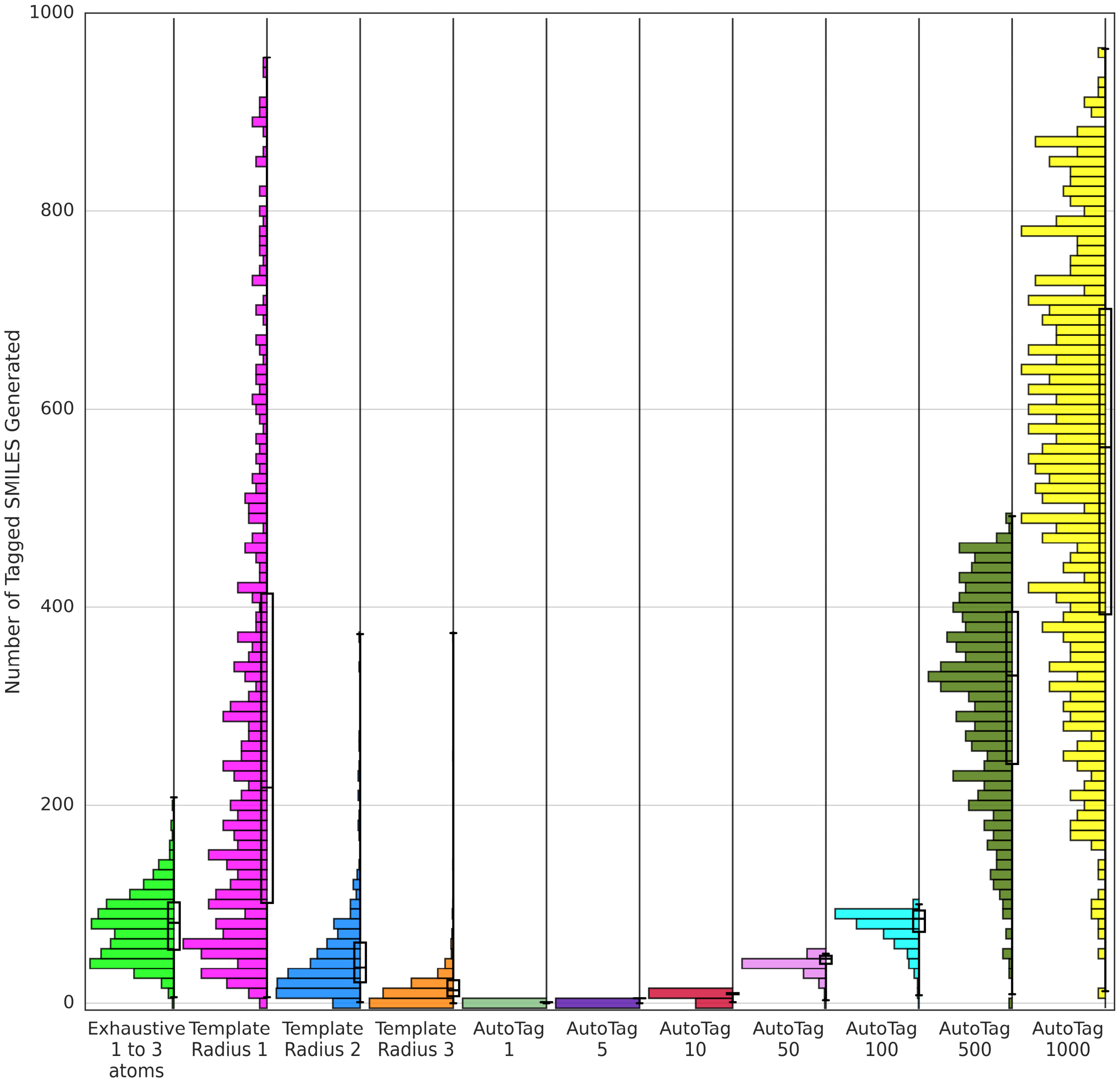

Figure S2. Number of tagged SMILES per molecule as function of the tagging method. The relative number of molecules (horizontal bar length) is plotted as function of the number of valid tagged SMILES per molecule (vertical axis) produced by different tagging methods (horizontal categories), tested over 500 molecules (randomly selected from the test set). A higher number of tags corresponds to a higher computational cost as each tagged starting material must be processed by the TTL.

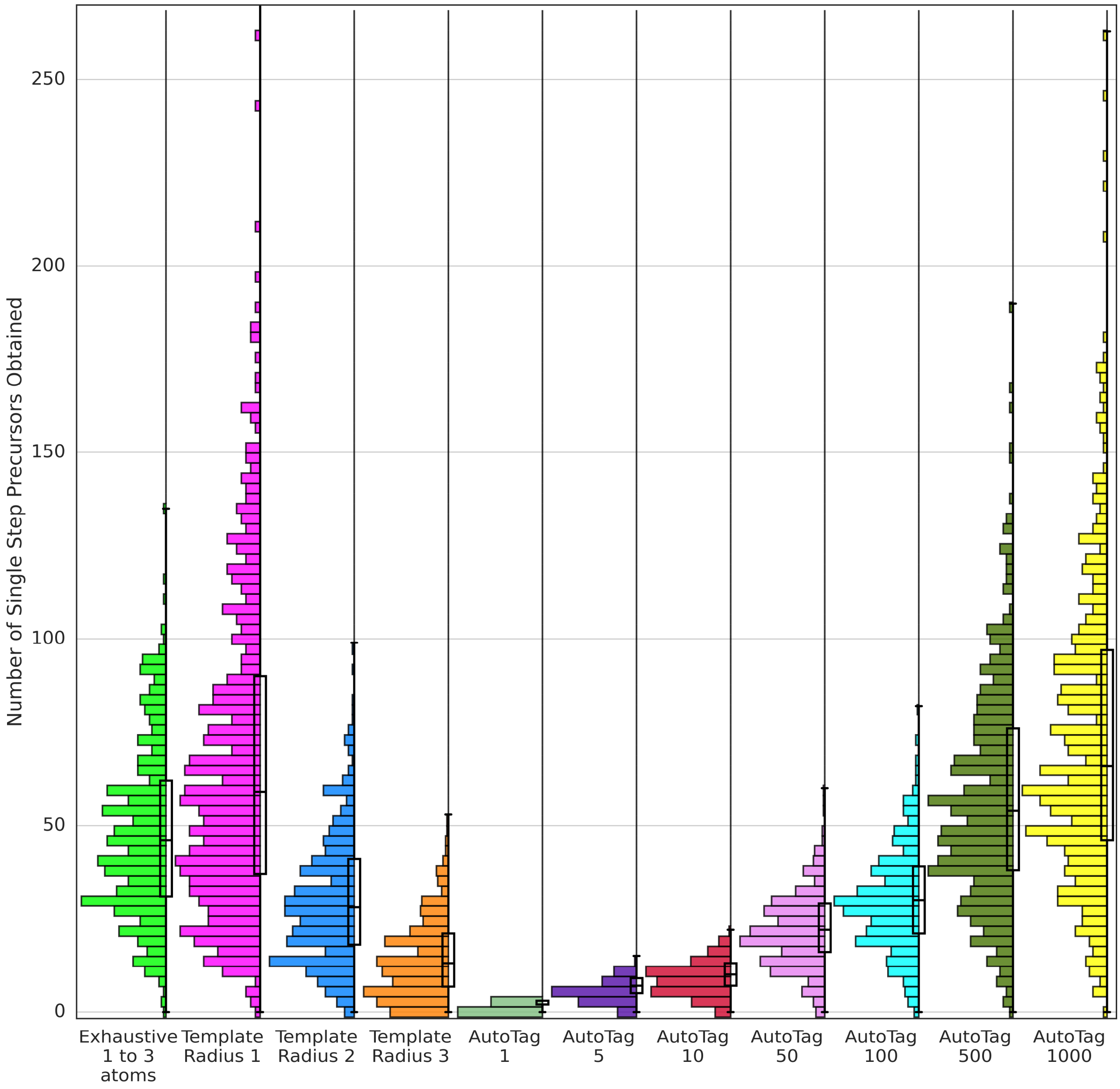

Figure S3. Number of starting materials per molecule from TTL as function of the tagging method. The relative number of molecules (horizontal bar length) is plotted a function of the number of starting materials per molecule (vertical axis, "single step precursors") produced by applying TTL to the tagged SMILES resulting from the indicated tagging method (horizontal categories), tested on 500 molecules (randomly selected from the test set) across multiple tagging strategies.

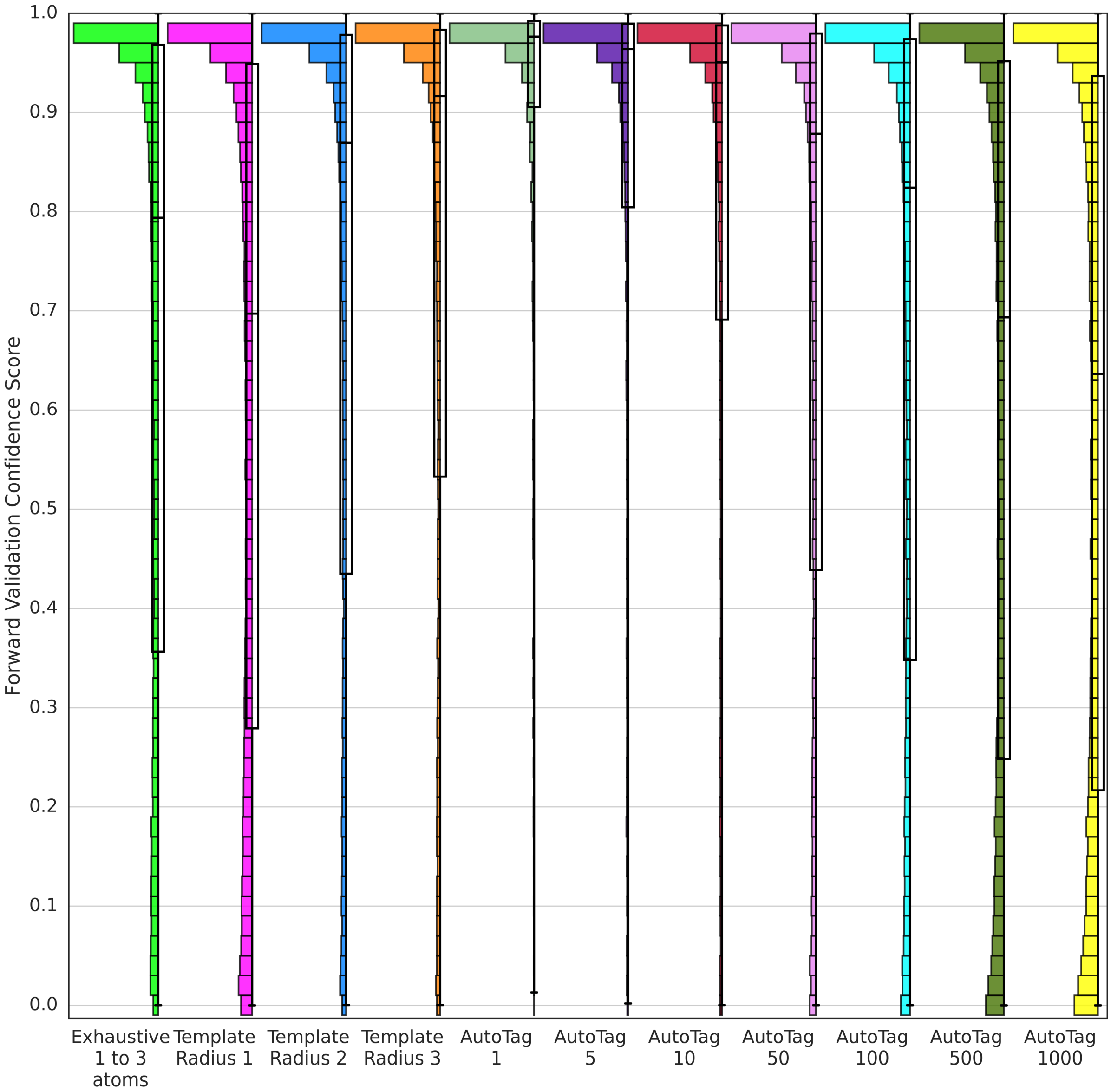

Figure S4. Distribution of forward validation confidence scores for validated TTL steps a function of the tagging method. The relative number of forward validated steps (horizontal bar length) is plotted as function of the confidence score of the forward validation transformer T3 (vertical axis) for steps predicted from SMILES tagged with different tagging methods (horizontal categories), tested over 500 molecules (randomly selected from the test set).

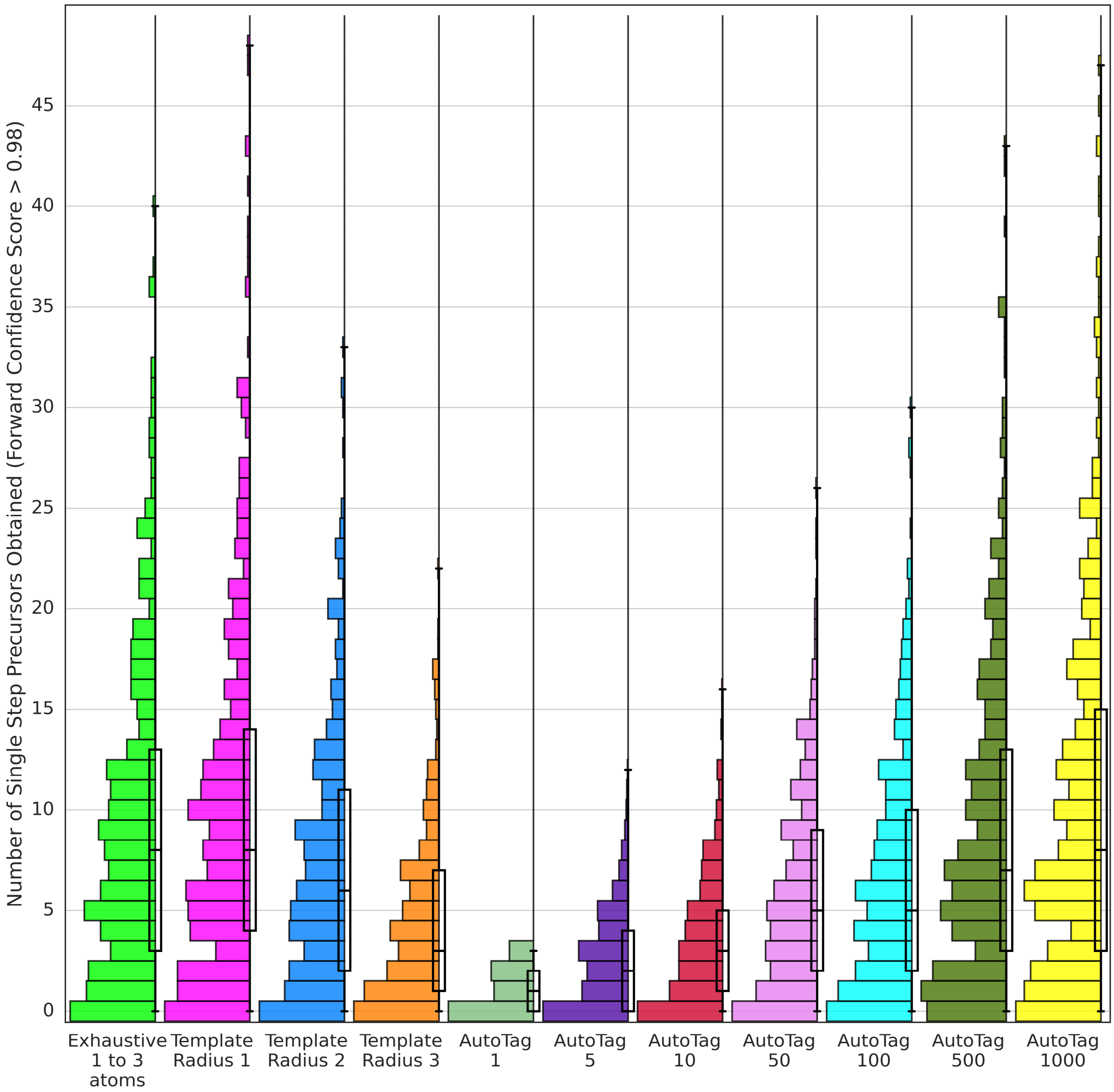

Figure S5. Number of single step precursors produced by TTL as function of the tagging method. The relative number of molecules (horizontal bar length) is plotted as function of the number of precursors obtained from validated TTL predicted single retrosynthetic steps per molecule (vertical axis) using different tagging methods (horizontal categories), tested on 500 molecules (randomly selected from the test set).

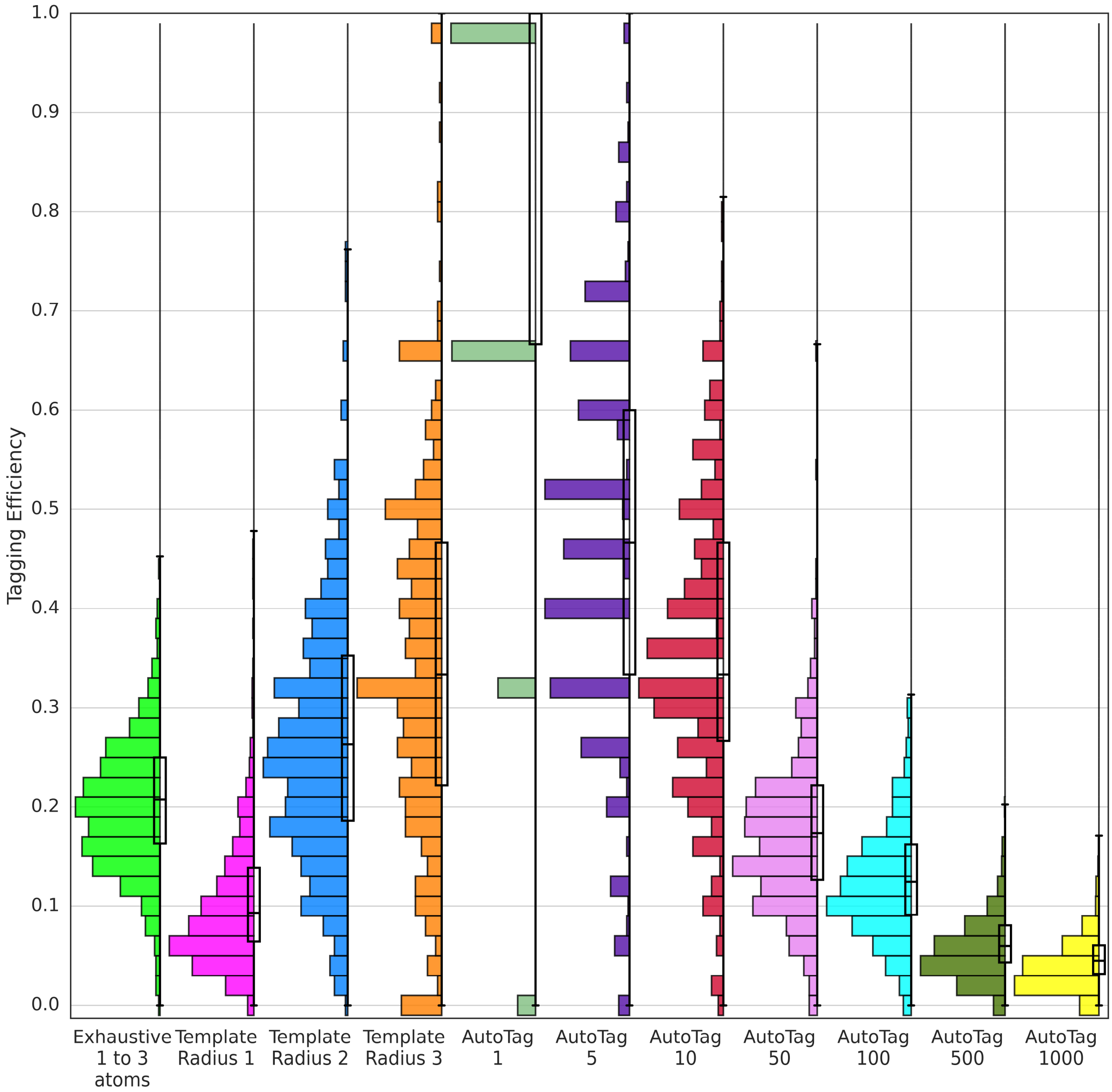

Figure S6. Tagging efficiency as function of the tagging method. The number of molecules (horizontal bar length) is plotted as function of the fraction of tags leading to a TTL validated retrosynthetic step (vertical axis) using different tagging methods (horizontal categories), tested over 500 molecules (randomly selected from the test set). The tagging efficiency was computed by dividing the number of TTL validated retrosyntheses obtained by the number of generated tagged SMILES. Values are normalized, predictions were obtained with a beam size of 3 for T2 (reagent prediction), all tested on the forward validation model T3.

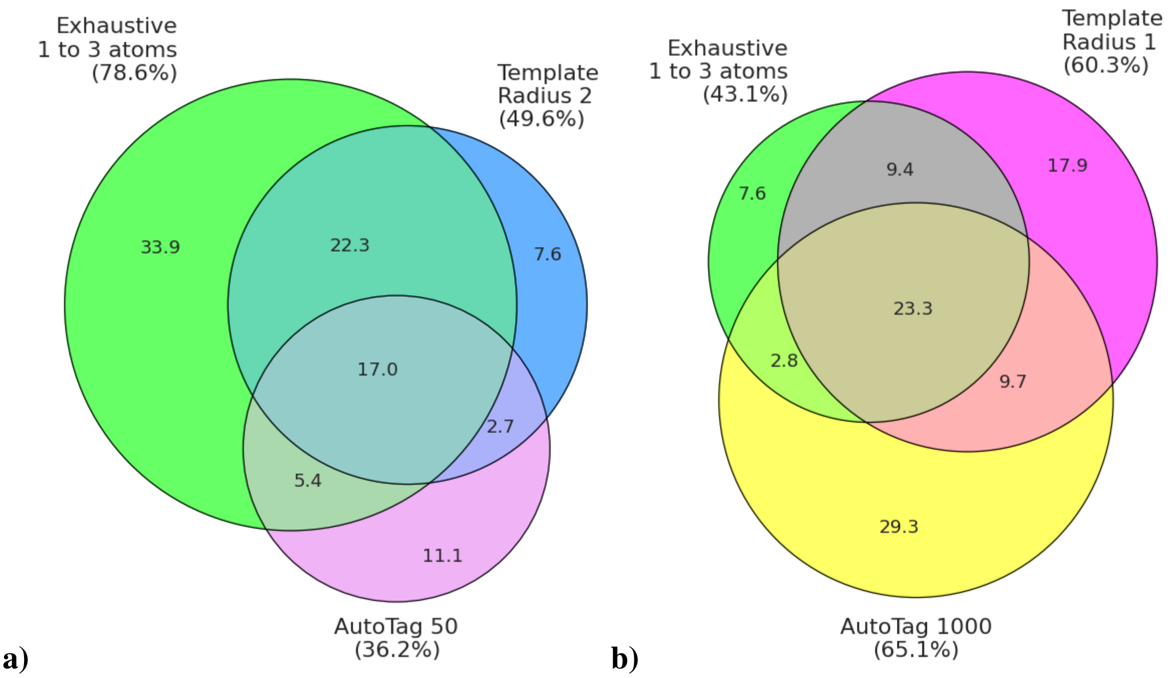

Figure S7. Overlap of retrosynthetic steps predicted by TTL using different tagging methods. The Venn diagram shows the percentage of TTL predicted steps distributed across three different tagging methods choses as (**a**) the selected set of reasonable tagging methods that avoids excessive number of tags, and (**b**) the three least restrictive tagging methods generating large number of tags (computationally expensive), tested over 500 molecules (randomly selected from the test set). Selection (**a**) is subsequently used for the multistep predictions in TTLA.

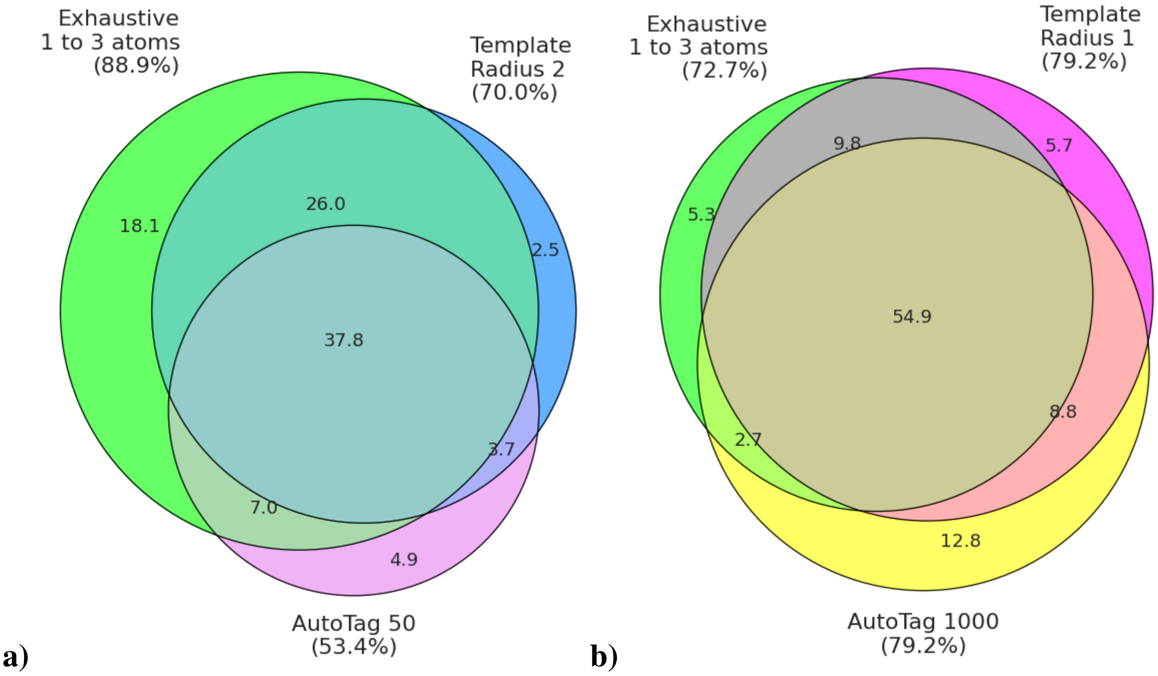

Figure S8. Overlap of high confidence retrosynthetic steps predicted by TTL using different tagging methods. Same analysis as Figure S7 for the subset of validated step having a confidence score higher than 98% for forward validation transformer T3.

Multistep predictions

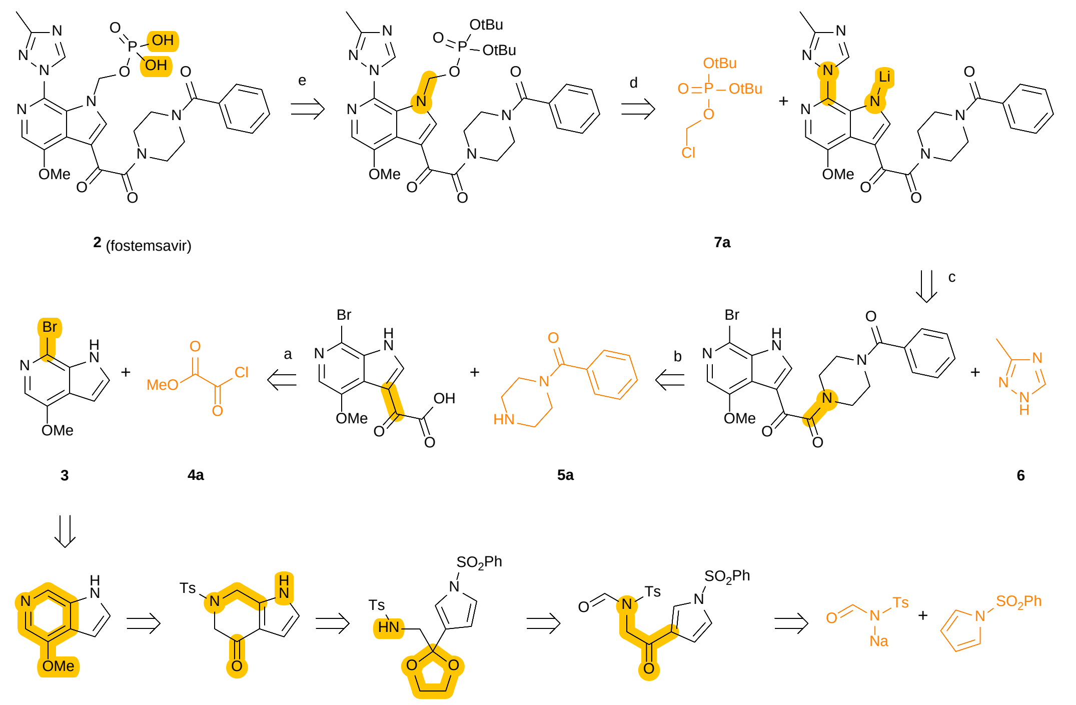

Figure S9. Literature reported retrosynthesis for fostemsavir.[40] Orange-coloured compounds are commercially available. Reported reagents: a) $AlCl_3$, Bu_4NHSO_4, CH_2Cl_2, then KOH, then H_3PO_4; b) Ph_2POCl, NMM, NMP; c) KOH, CuI, then KOH, EtOH, LiI; d) Et_4NI, K_2CO_3, CH_3CN/H_2O; e) AcOH, H_2O.

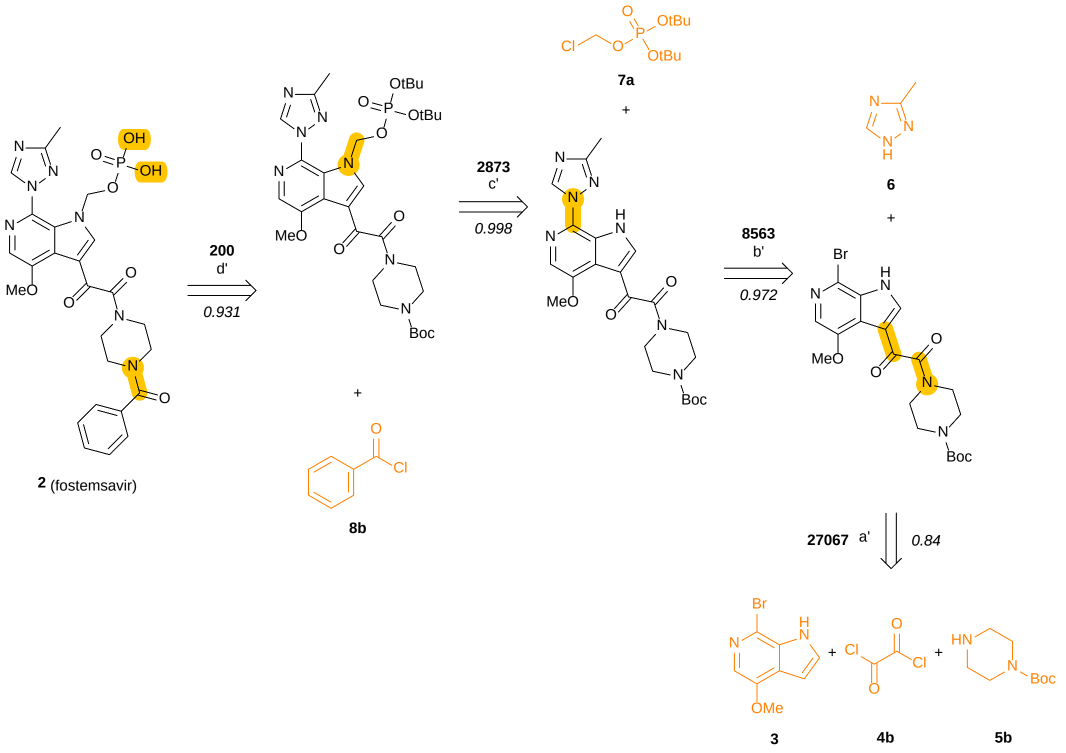

Figure S10. Best RPScoring predicted retrosynthesis route for fostemsavir. Orange-coloured compounds are commercially available. Except for some of the commercial precursors that were present but involved in different reactions, none of the intermediate compounds were present in the training dataset. The reaction prediction numbers in bold on retrosynthesis arrows correspond to the order in

which the multistep tree search prioritized the prediction. Forward prediction confidence scores are shown under retrosynthesis arrows. Predicted reaction conditions: a‘) Et_3N, CH_2Cl_2; b’) K_2CO_3, CuI, toluene; c’) K_2CO_3, DMF; d’) HCl, N,N-Diisopropylethylamine, H_2O, dioxane.

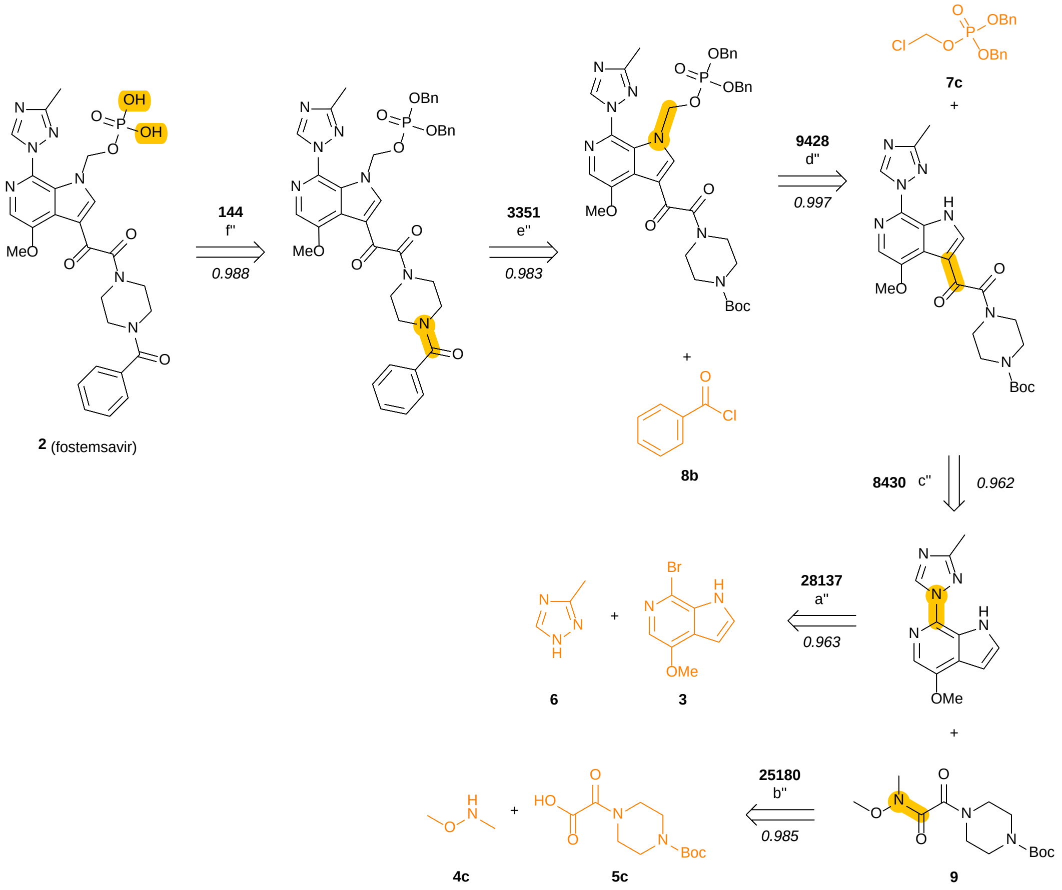

Figure S11. Best overall confidence score predicted retrosynthesis route for fostemsavir. Orange-coloured compounds are commercially available. Except for some of the commercial precursors that were present but involved in different reactions, none of the intermediate compounds were present in the training dataset. The reaction prediction numbers in bold on retrosynthesis arrows correspond to the order in which the multistep tree search prioritized the prediction. Forward prediction confidence scores are shown under retrosynthesis arrows. Predicted reagents: a”) (2S)-pyrrolidine-2-carboxylic acid, K_2CO_3, CuI, EtOAc, DMSO; b”) no reagent predicted; c”) *n*-BuLi, THF; d”) K_2CO_3, DMF; e”) TFA, DMAP, CH_2Cl_2, f”) Pd, EtOH.

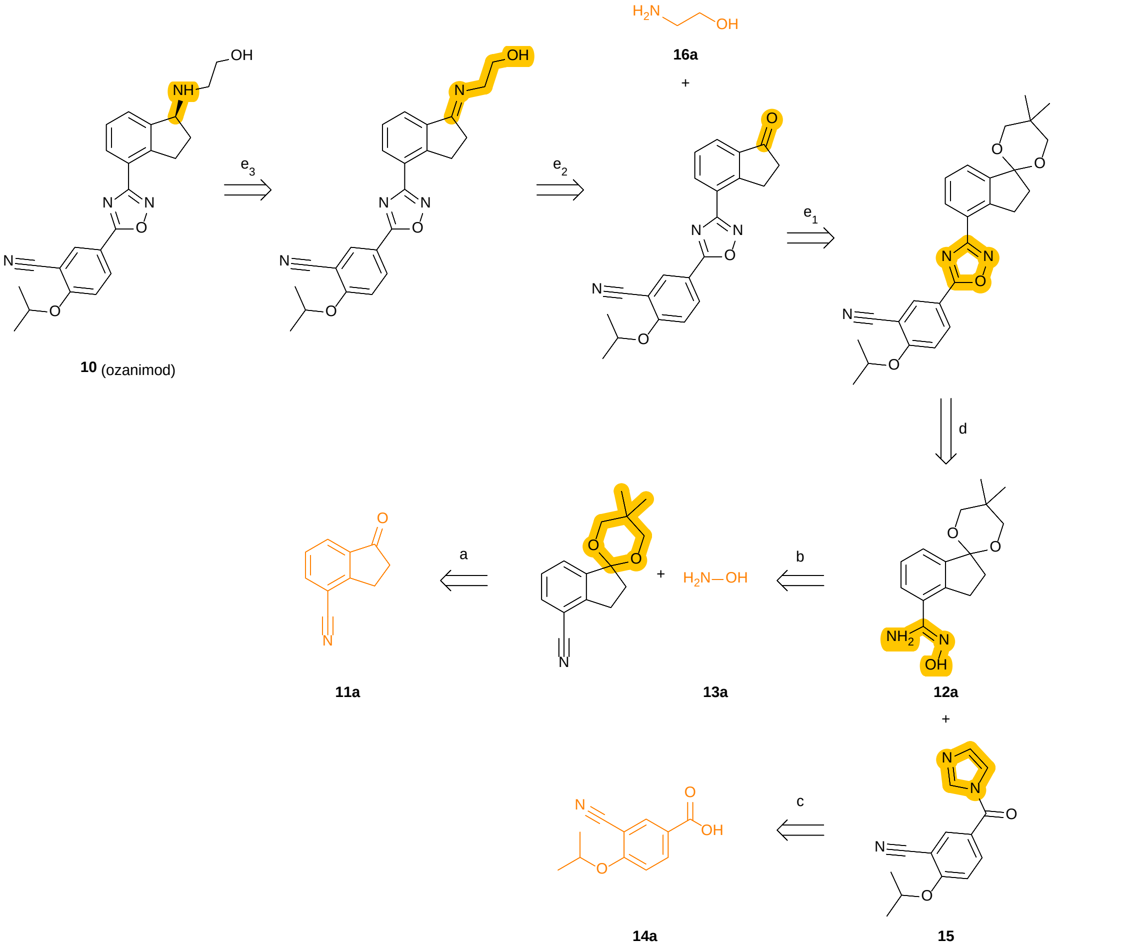

Figure S12. Literature reported retrosynthesis for ozanimod.[40] Orange-coloured compounds are commercially available. Reported reagents: a) HC(Ome)$_3$, *p*-TsOH, PhCH$_3$; b) NH_2OH.HCl, Et$_3$N; c) carbonyl diimidazole; d) NaOH; e) i) *p*-TsOH, acetone, ii) $NH_2CH_2CH_2OH$, *p*-TsOH, PhCH$_3$, iii) Chiral Ru-complex, Et$_3$N/HCO$_2$H.

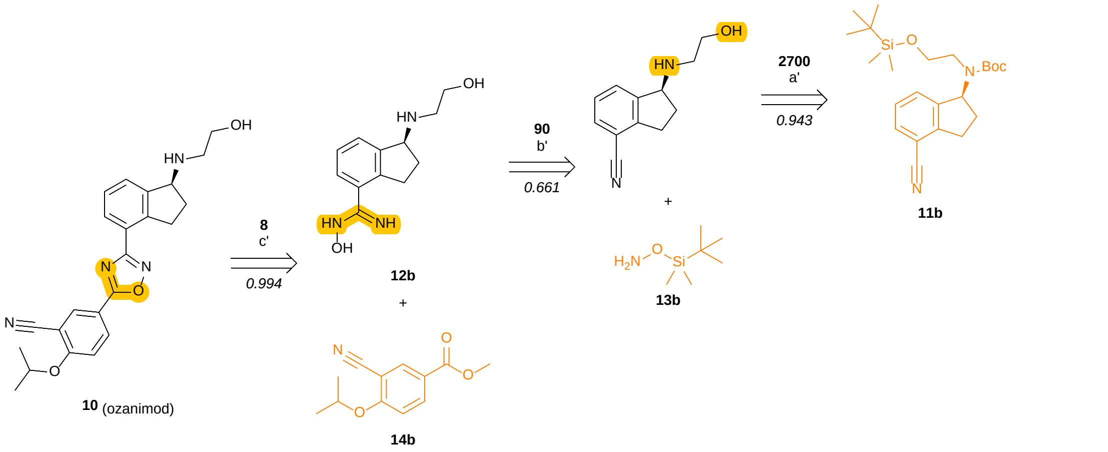

Figure S13. Best RPScoring predicted retrosynthesis route for ozanimod. Orange-coloured compounds are commercially available. Except for some of the commercial precursors that were present but involved in different reactions, none of the intermediate compounds were present in the training dataset. The reaction prediction numbers in bold on retrosynthesis arrows correspond to the order in which the multistep tree search prioritized the prediction. Forward prediction confidence scores are shown under retrosynthesis arrows. Predicted reagents: a') HCl, dioxane; b') ZnCl$_2$, AcOEt, toluene; c') HCl, *t*-BuOK, THF.

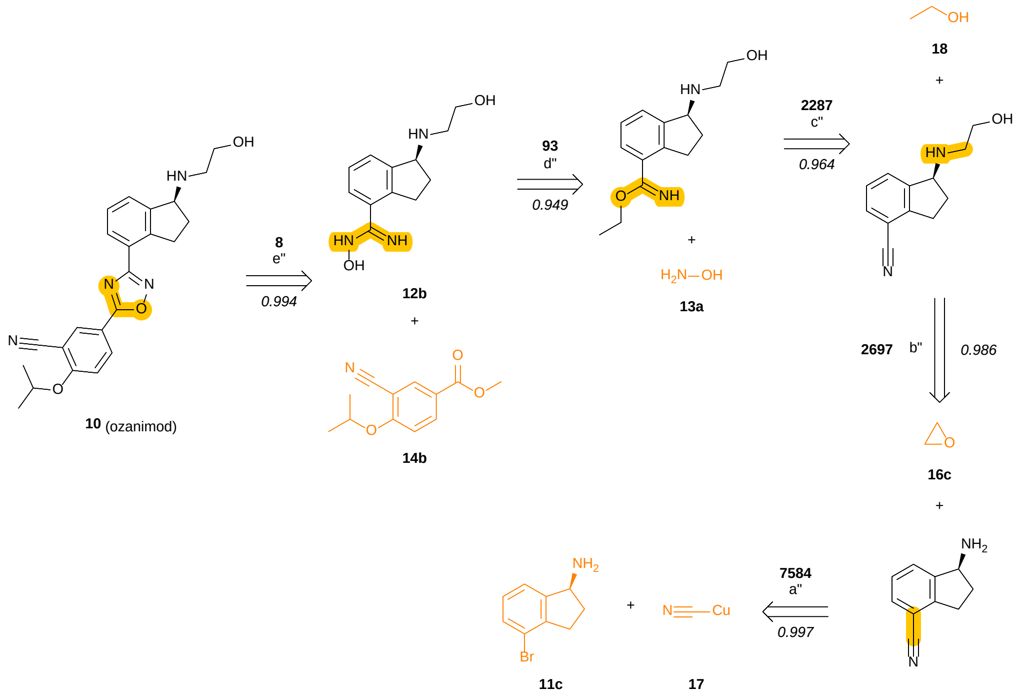

Figure S14. Best overall confidence score predicted retrosynthesis route for ozanimod. Orange-coloured compounds are commercially available. Except for some of the commercial precursors that were present but involved in different reactions, none of the intermediate compounds were present in the training dataset. The reaction prediction numbers in bold on retrosynthesis arrows correspond to the order in which the multistep tree search prioritized the prediction. Forward prediction confidence scores are shown under retrosynthesis arrows. Predicted reagents: a") 1-Methylpyrrolidin-2-one; b") no reagent predicted; c") HCl, Et_2O; d") HCl, $NaHCO_3$, EtOH; e") HCl, *t*-BuOK, THF.

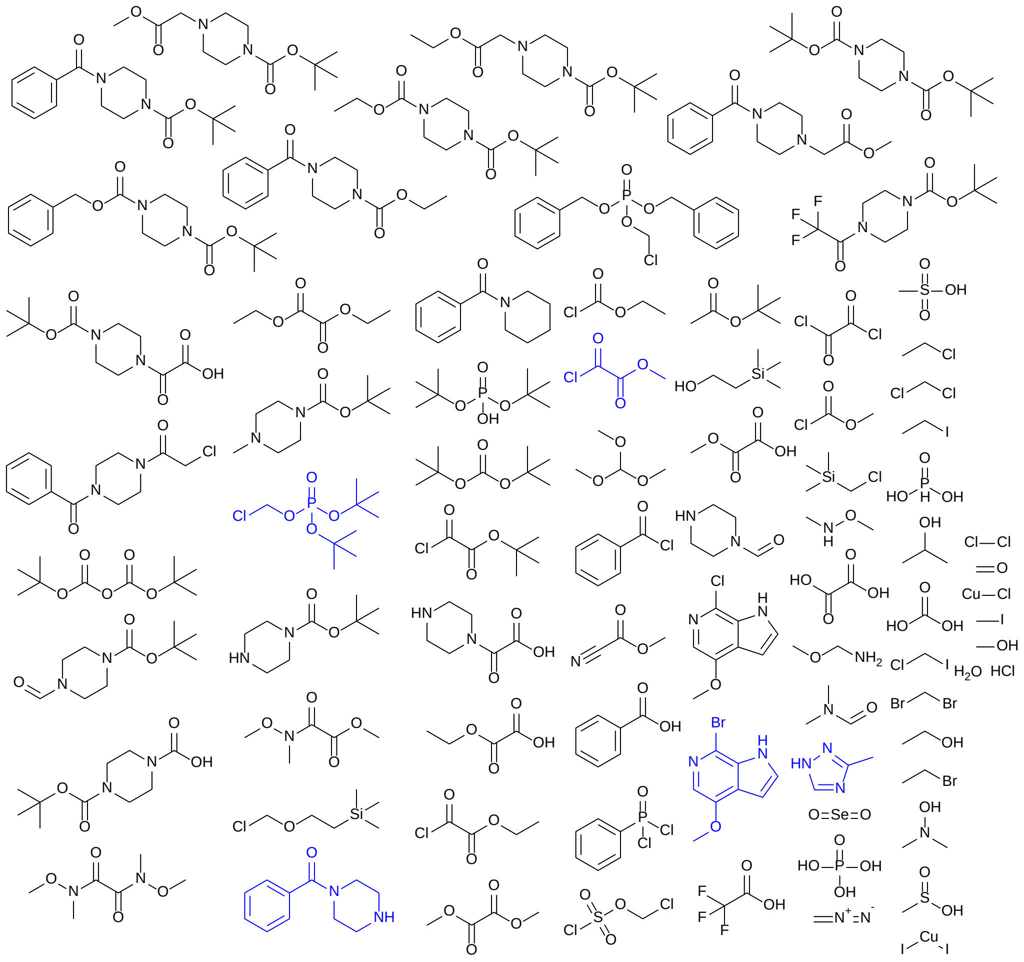

Figure S15. Set of commercially available precursors of all solved routes for fostemsavir. All building blocks of the literature reported retrosynthesis are highlighted in blue.

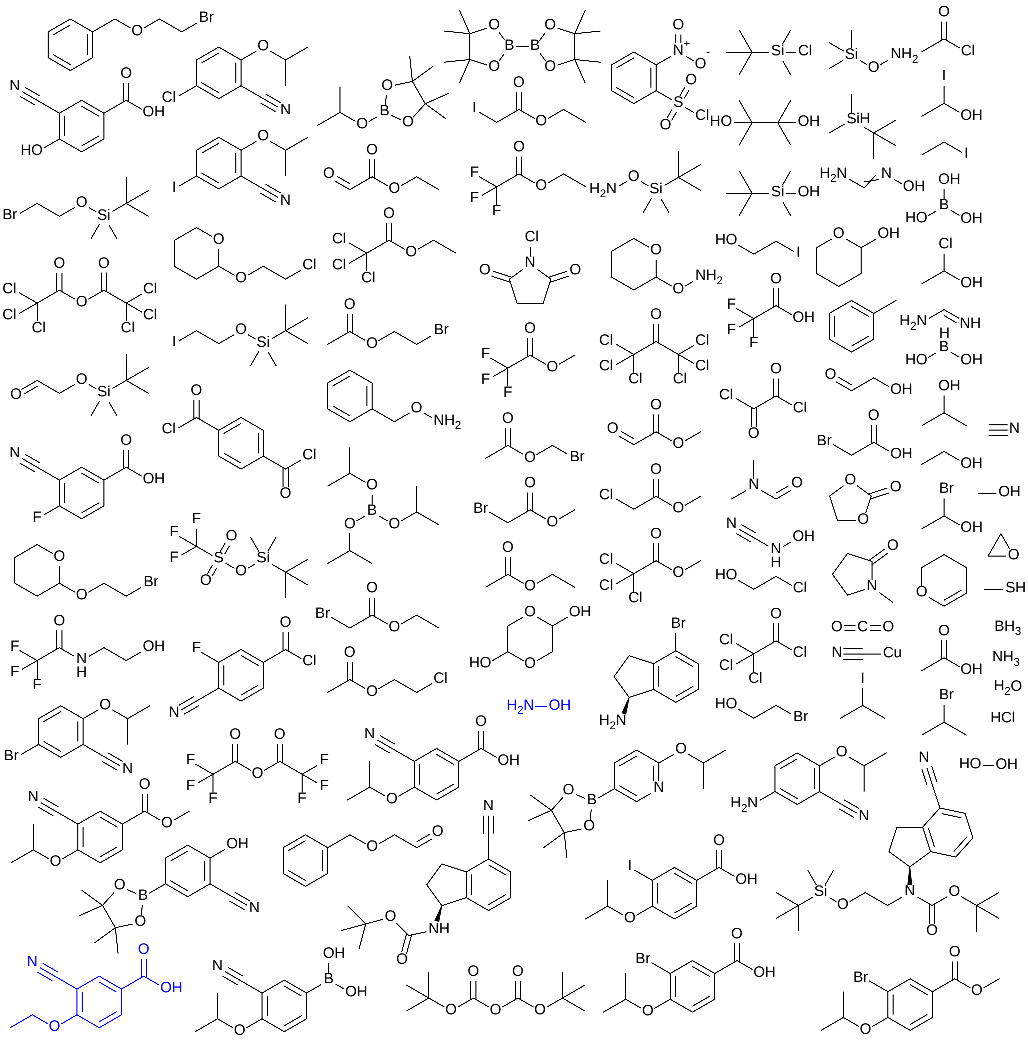

Figure S16. Set of commercially available precursors of all solved routes for ozanimod. Some of the building blocks of the literature reported retrosynthesis are highlighted in blue.

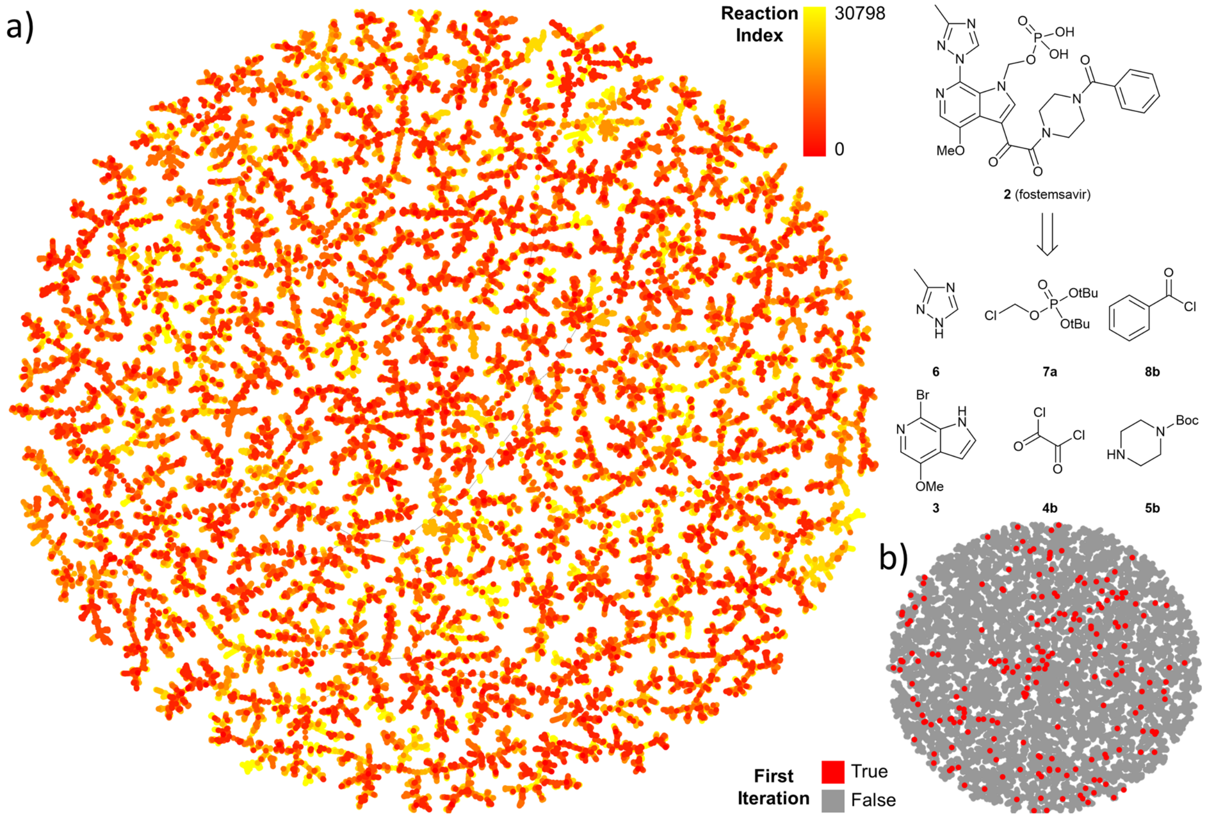

Figure S17. TMAP representation of iterated predictions for the multistep search of fostemsavir. (**a**) Predicted reactions from the target molecule (low indexes) to end nodes. (**b**) Highlighted first iteration of the TTLA search. Interactive map available at https://tm.gdb.tools/TTLA/fostemsavir.

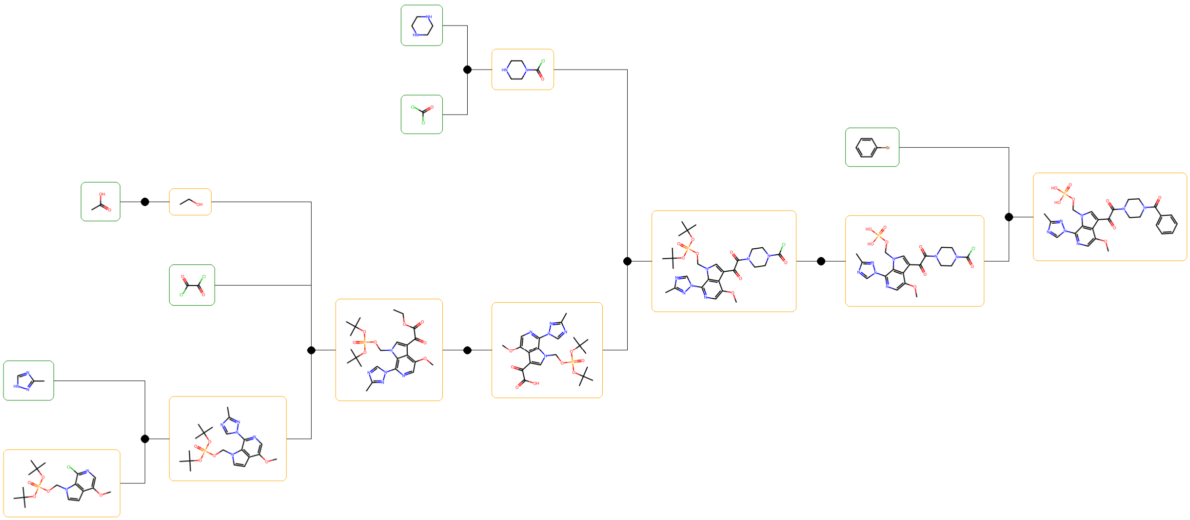

Figure S18. Fostemsavir retrosynthesis route predicted by AiZynthFinder (v3.7.0).

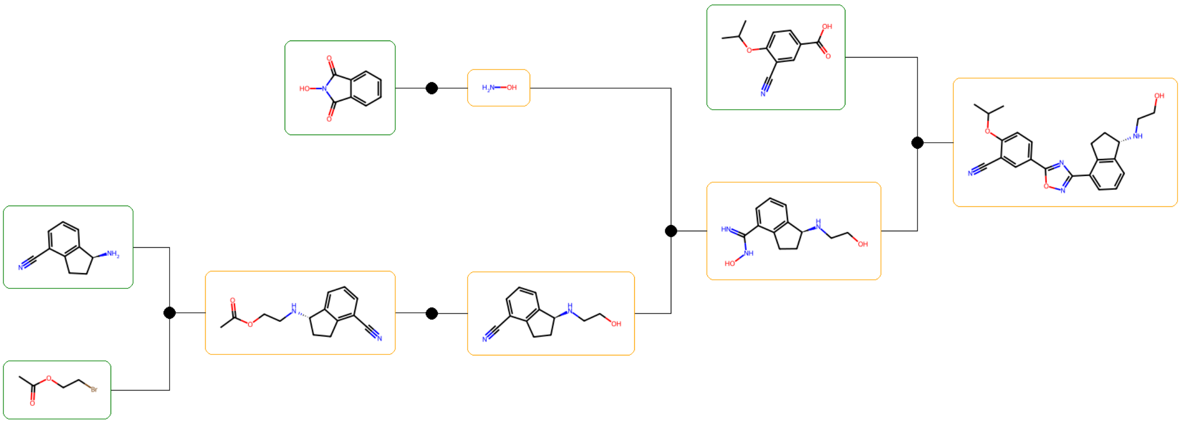

Figure S19. Ozanimod retrosynthesis route predicted by AiZynthFinder (v3.7.0).

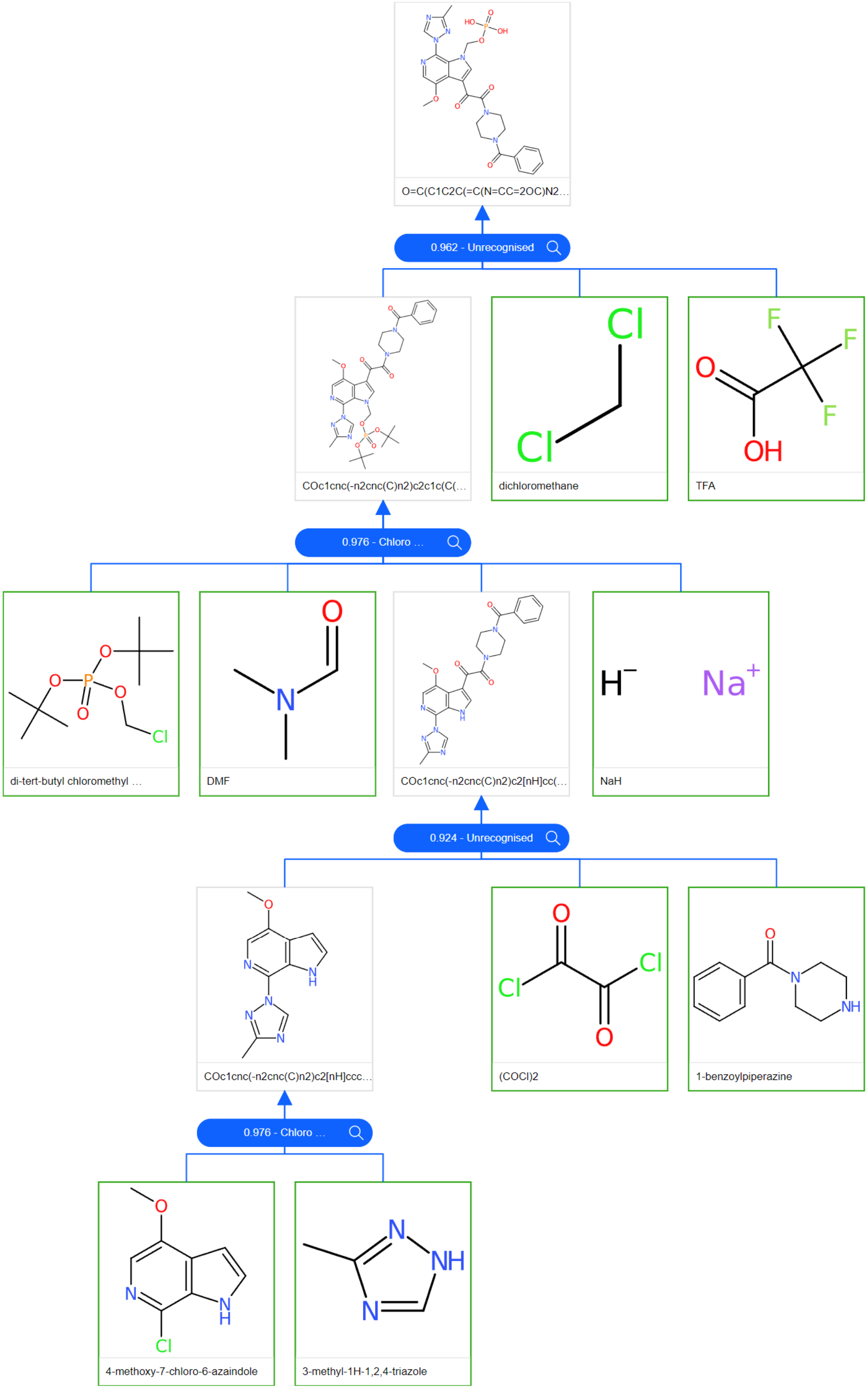

Figure S20. Fostemsavir retrosynthesis route predicted by IBM RXN for Chemistry user interface using the default “12class-tokens-2021-05-14” models, with highest quality tuning, and excluding commercially similar compounds as in our route prediction settings.

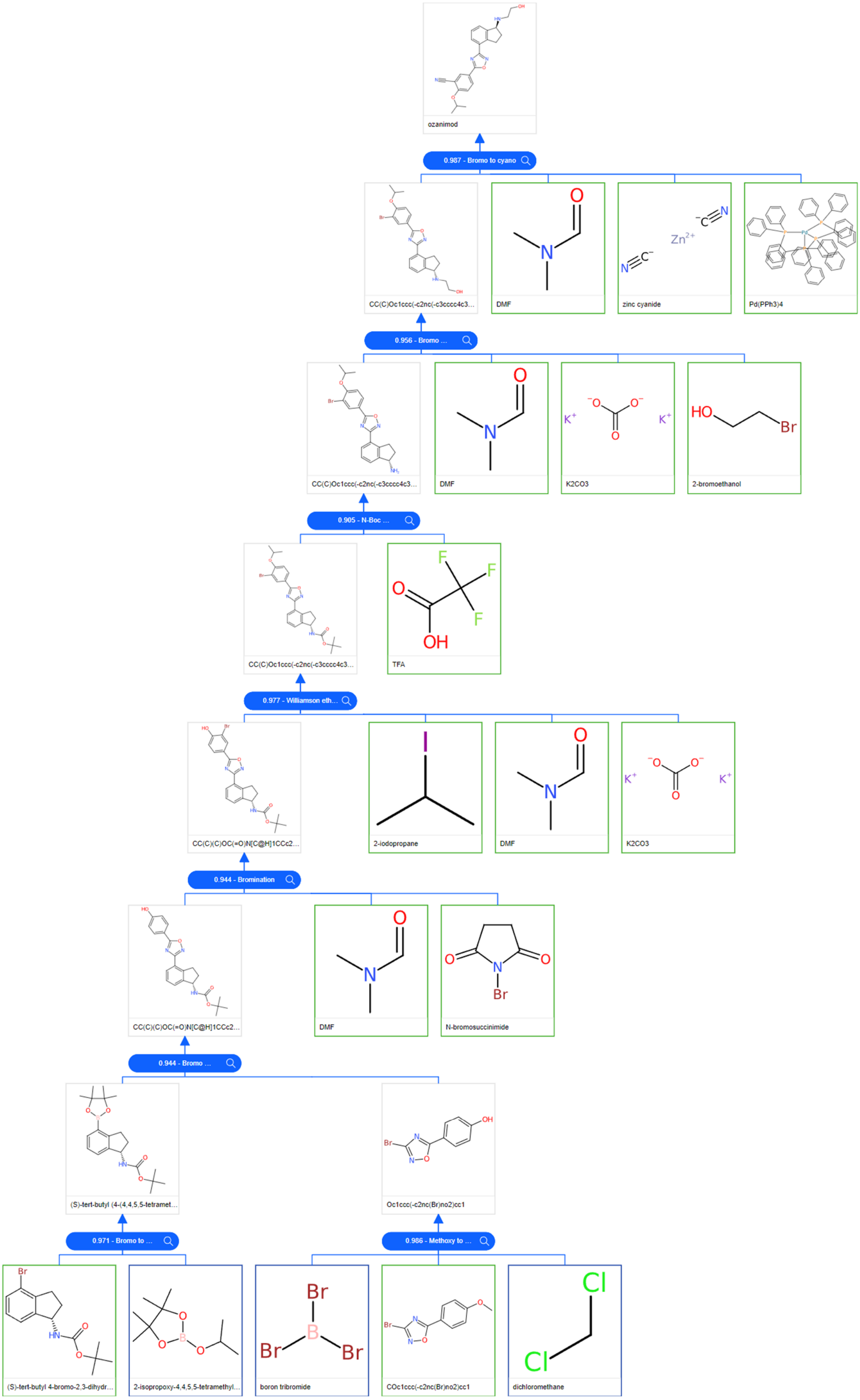

Figure S21. Ozanimod retrosynthesis route predicted by IBM RXN for Chemistry user interface using the default "12class-tokens-2021-05-14" models, with highest quality tuning, and excluding commercially similar compounds as in our route prediction settings.

Target SMILES: CCCCc1nn(-c2cc(NC(=O)CC)ccc2Cl)c(C#N)c1Cc1ccc(Br)cc1F

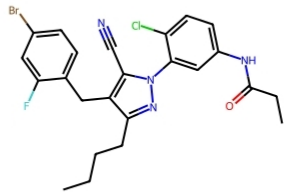

Overall forward confidence score = 0.6502
Overall Guiding RPScore = 0.025
Overall Penalties = 0.0938
Number of steps = 5

Best RPScore route:

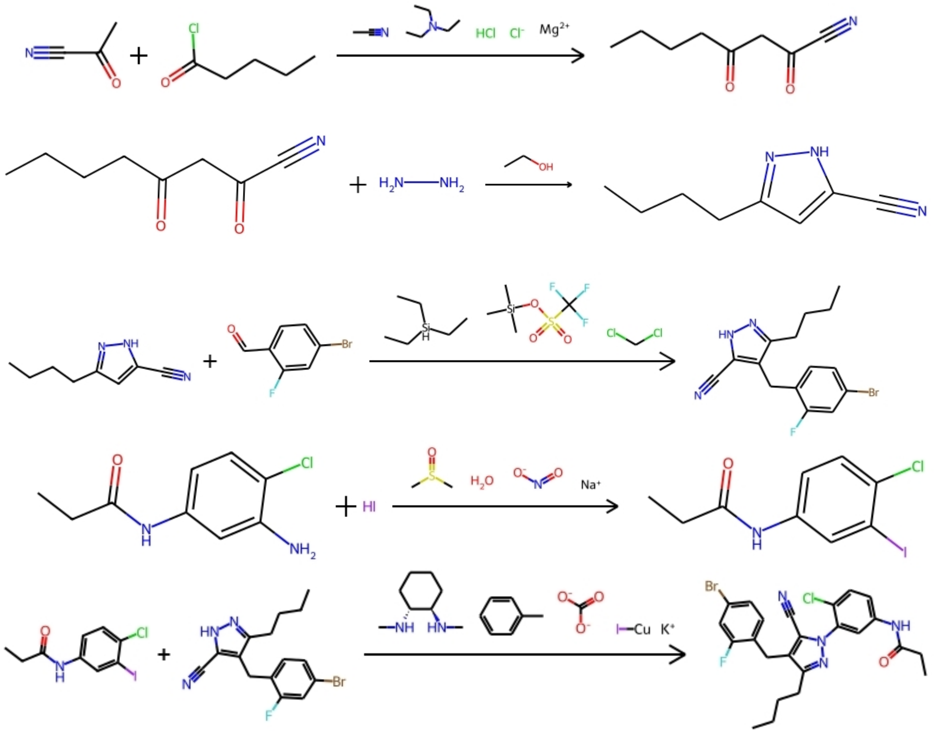

Figure S22. Best RPScoring predicted route by our TTLA. Target molecule selected from the benchmark of Genheden *et al*., see main text.

Target SMILES: CCOC(=O)N1CCc2c(c3cccc(N)c3n2C)C1

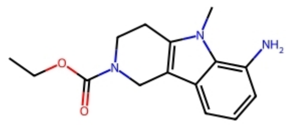

Overall forward confidence score = 0.7725
Overall Guiding RPScore = 0.2858
Overall Penalties = 0.4625
Number of steps = 2

Best RPScore route:

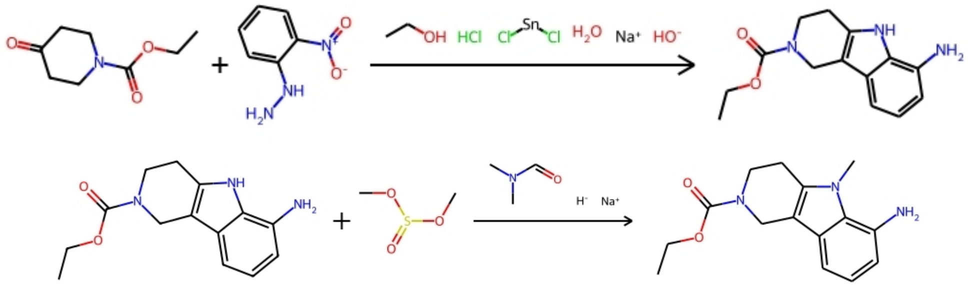

Figure S23. Best RPScoring predicted route by our TTLA. Target molecule selected from the benchmark of Genheden *et al*., see main text.

Target SMILES: COc1ccc(CCNc2nccc(-c3cccc(NC(C)=O)c3)n2)cc1OC

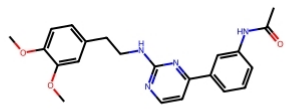

Overall forward confidence score = 0.8665
Overall Guiding RPScore = 0.4439
Overall Penalties = 0.6403
Number of steps = 2

Best RPScore route:

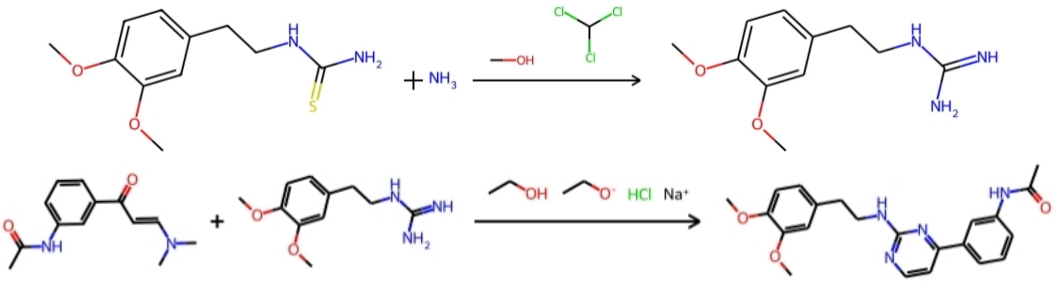

Figure S24. Best RPScoring predicted route by our TTLA. Target molecule selected from the benchmark of Genheden *et al*., see main text.

Target SMILES: CCCCc1nc(-c2ccc(F)cc2)c(C(=O)N(CC)CC)n1Cc1ccc(-c2ccccc2C#N)cc1

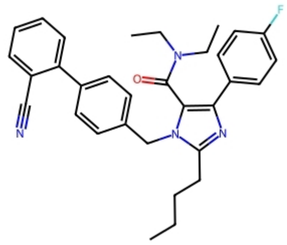

Overall forward confidence score = 0.8949
Overall Guiding RPScore = 0.0795
Overall Penalties = 0.1389
Number of steps = 3

Best RPScore route:

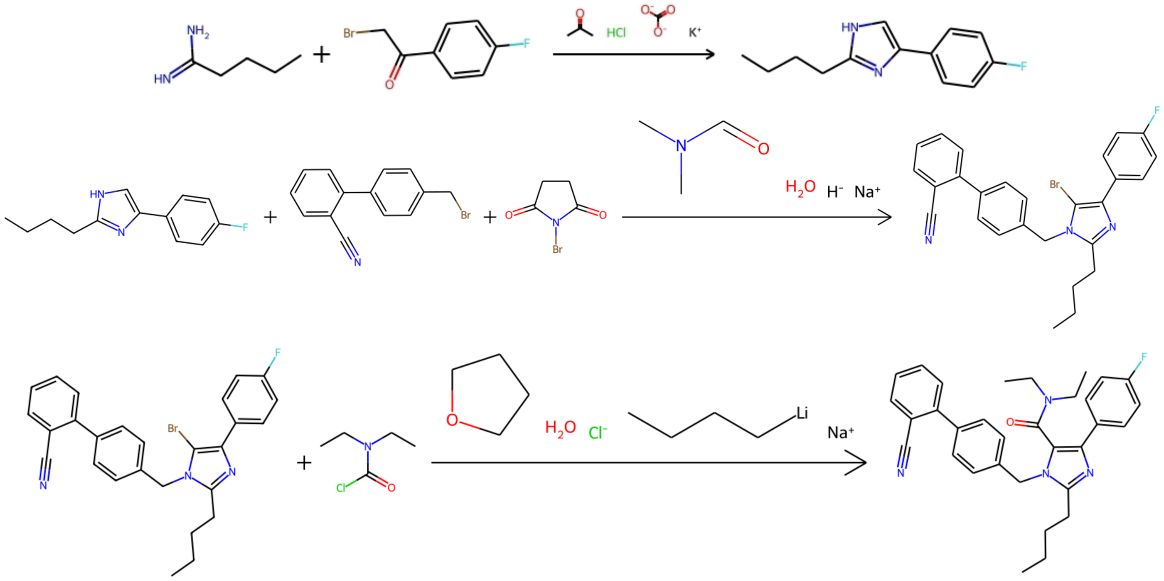

Figure S25. Best RPScoring predicted route by our TTLA. Target molecule selected from the benchmark of Genheden *et al*., see main text.

Target SMILES: O=C(O)C1CN(Cc2ccc(-c3noc(CCC4(c5ccccc5)CCCCC4)n3)cc2)C1

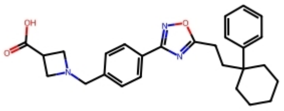

Overall forward confidence score = 0.7397
Overall Guiding RPScore = 0.1437
Overall Penalties = 0.3035
Number of steps = 3

Best RPScore route:

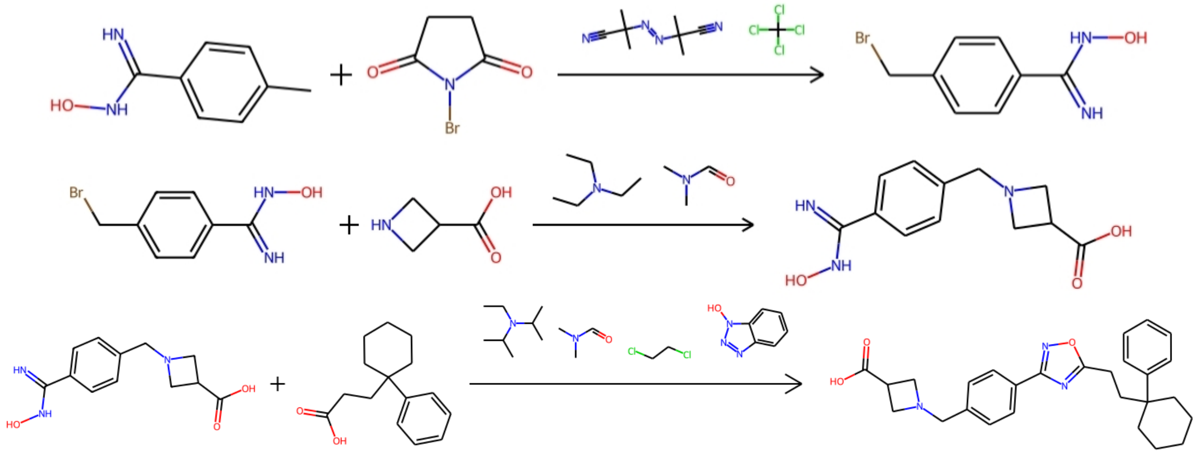

Figure S26. Best RPScoring predicted route by our TTLA. Target molecule selected from the benchmark of Genheden *et al.*, see main text.

Target SMILES: Cc1ccc(-n2ccc(C(F)(F)F)n2)nc1Cl

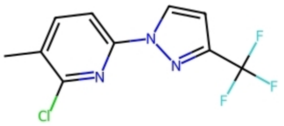

Overall forward confidence score = 0.9783
Overall Guiding RPScore = 0.6513
Overall Penalties = 0.8323
Number of steps = 2

Best RPScore route:

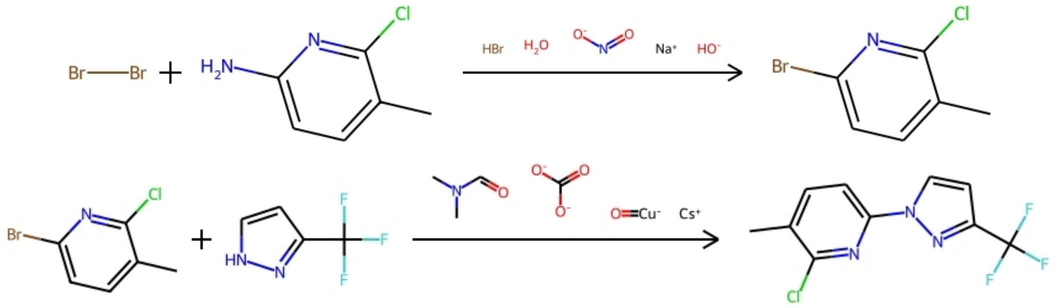

Figure S27. Best RPScoring predicted route by our TTLA. Target molecule selected from the benchmark of Genheden *et al.*, see main text.

Target SMILES: O=C(Nc1ccc2c(CN3CCCC3)cn(Cc3c(Cl)cccc3Cl)c2c1)Oc1ccccc1

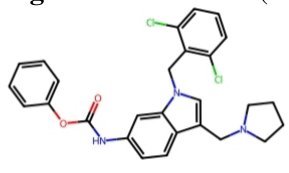

Overall forward confidence score = 0.5652
Overall Guiding RPScore = 0.1587
Overall Penalties = 0.351
Number of steps = 2

Best RPScore route:

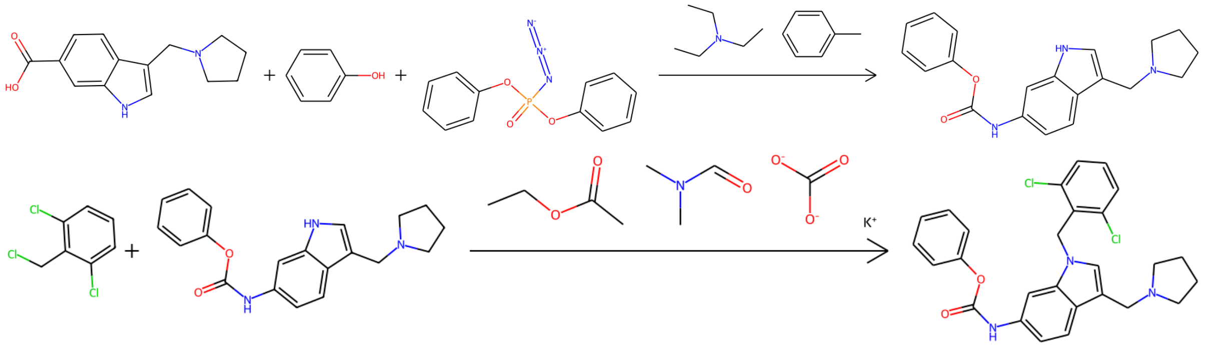

Figure S28. Best RPScoring predicted route by our TTLA. Target molecule selected from the benchmark of Genheden *et al.*, see main text.

Target SMILES: c1ccc2oc(-c3ccc4c(c3)[nH]c3ccccc34)nc2c1

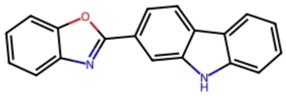

Overall forward confidence score = 0.8932
Overall Guiding RPScore = 0.8932
Overall Penalties = 1.0
Number of steps = 1

Best RPScore route:

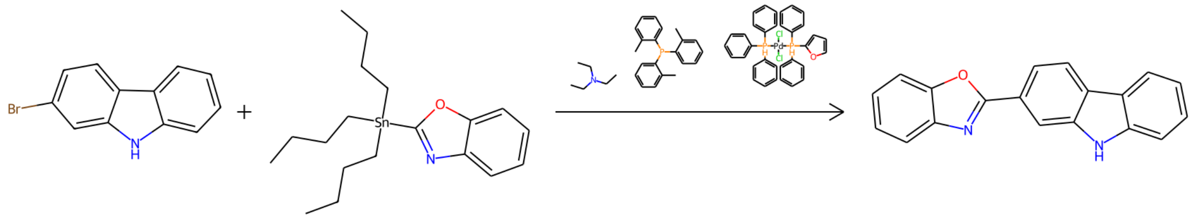

Figure S29. Best RPScoring predicted route by our TTLA. Target molecule selected from the benchmark of Genheden *et al.*, see main text.

Target SMILES: COc1ccc(C2(c3cccc(OCCCF)c3)N=C(N)c3ncccc32)cc1C(F)(F)F

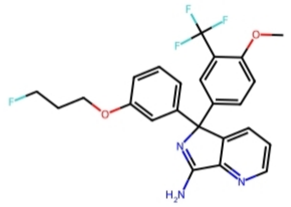

Overall forward confidence score = 0.4259
Overall Guiding RPScore = 0.0892
Overall Penalties = 0.3272
Number of steps = 3

Best RPScore route:

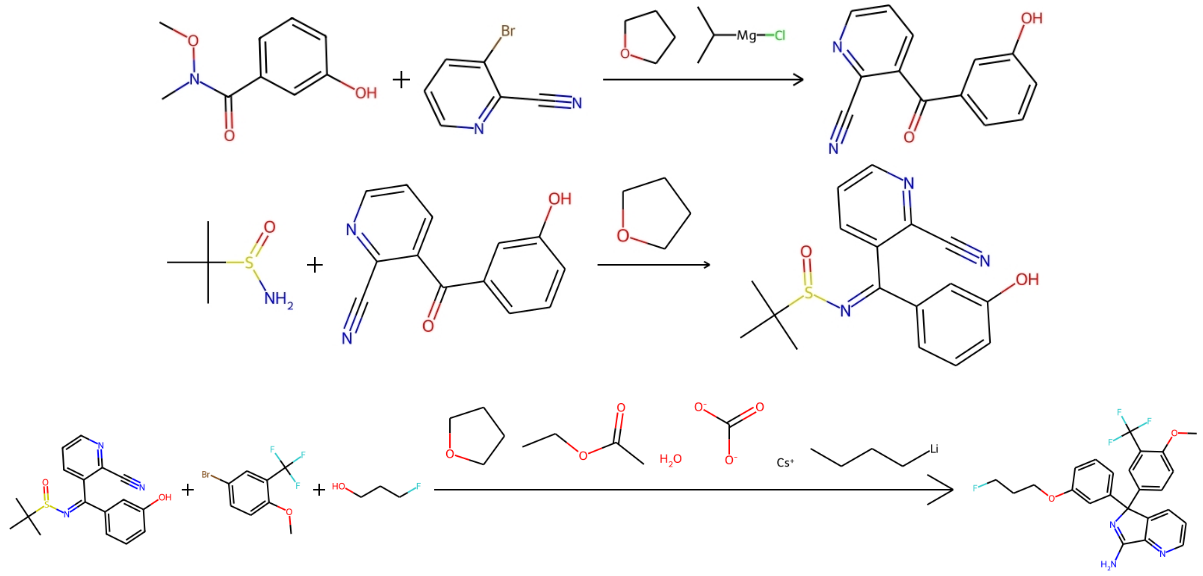

Figure S30. Best RPScoring retrosynthesis route predicted by our TTLA. Target molecule selected from the benchmark of Genheden *et al.*, see main text.

Target SMILES: CS(=O)(=O)c1ccc(C2=C(c3ccc(-c4ccsc4)cc3)C(=O)OC2)cc1

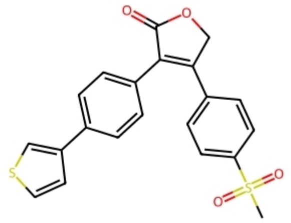

Overall forward confidence score = 0.7994
Overall Guiding RPScore = 0.3674
Overall Penalties = 0.5744
Number of steps = 2

Best RPScore route:

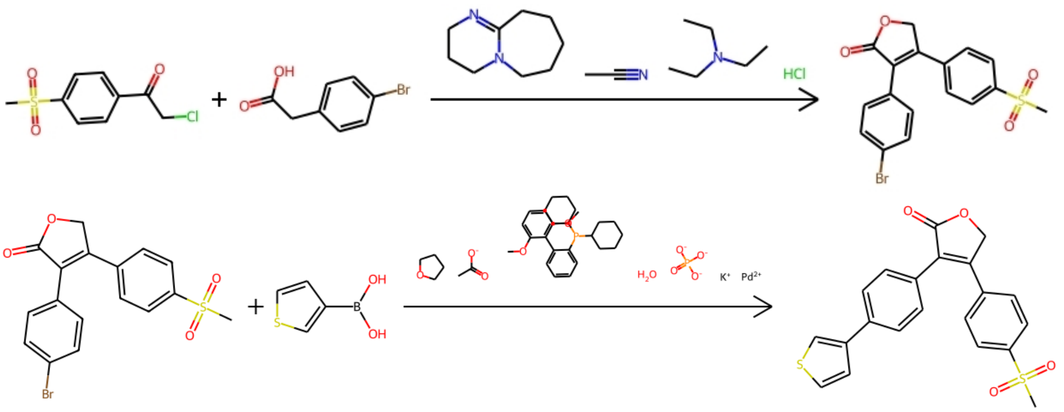

Figure S31. Best RPScoring retrosynthesis route predicted by our TTLA. Target molecule selected from the benchmark of Genheden *et al.*, see main text.
